# Single-cell morphological characterization of CRH neurons throughout the whole mouse brain

**DOI:** 10.1186/s12915-021-00973-x

**Published:** 2021-03-15

**Authors:** Yu Wang, Pu Hu, Qinghong Shan, Chuan Huang, Zhaohuan Huang, Peng Chen, Anan Li, Hui Gong, Jiang-Ning Zhou

**Affiliations:** 1 Chinese Academy of Science Key Laboratory of Brain Function and Diseases, School of Life Sciences, Division of Life Sciences and Medicine, University of Science and Technology of China, Hefei, 230026 China; 2 Center for Excellence in Brain Science and Intelligence Technology, Chinese Academy of Sciences, Shanghai, 200031 China; 3 Britton Chance Center for Biomedical Photonics, Wuhan National Laboratory for Optoelectronics, MoE Key Laboratory for Biomedical Photonics, School of Engineering Sciences, Huazhong University of Science and Technology, Wuhan, 430074 China

**Keywords:** Corticotropin-releasing hormone, fMOST imaging, Dendritic morphology, Three-dimensional reconstruction, Local circuit, Dendritic varicosities, Dendritic spine

## Abstract

**Background:**

Corticotropin-releasing hormone (CRH) is an important neuromodulator that is widely distributed in the brain and plays a key role in mediating stress responses and autonomic functions. While the distribution pattern of fluorescently labeled CRH-expressing neurons has been studied in different transgenic mouse lines, a full appreciation of the broad diversity of this population and local neural connectivity can only come from integration of single-cell morphological information as a defining feature. However, the morphologies of single CRH neurons and the local circuits formed by these neurons have not been acquired at brain-wide and dendritic-scale levels.

**Results:**

We screened the EYFP-expressing CRH-IRES-Cre;Ai32 mouse line to reveal the morphologies of individual CRH neurons throughout the whole mouse brain by using a fluorescence micro-optical sectioning tomography (fMOST) system. Diverse dendritic morphologies and projection fibers of CRH neurons were found in various brain regions. Follow-up reconstructions showed that hypothalamic CRH neurons had the smallest somatic volumes and simplest dendritic branches and that CRH neurons in several brain regions shared a common bipolar morphology. Further investigations of local CRH neurons in the medial prefrontal cortex unveiled somatic depth-dependent morphologies of CRH neurons that exhibited three types of mutual connections: basal dendrites (upper layer) with apical dendrites (layer 3); dendritic-somatic connections (in layer 2/3); and dendritic-dendritic connections (in layer 4). Moreover, hypothalamic CRH neurons were classified into two types according to their somatic locations and characteristics of dendritic varicosities. Rostral-projecting CRH neurons in the anterior parvicellular area had fewer and smaller dendritic varicosities, whereas CRH neurons in the periventricular area had more and larger varicosities that were present within dendrites projecting to the third ventricle. Arborization-dependent dendritic spines of CRH neurons were detected, among which the most sophisticated types were found in the amygdala and the simplest types were found in the hypothalamus.

**Conclusions:**

By using the CRH-IRES-Cre;Ai32 mouse line and fMOST imaging, we obtained region-specific morphological distributions of CRH neurons at the dendrite level in the whole mouse brain. Taken together, our findings provide comprehensive brain-wide morphological information of stress-related CRH neurons and may facilitate further studies of the CRH neuronal system.

**Supplementary Information:**

The online version contains supplementary material available at 10.1186/s12915-021-00973-x.

## Background

Corticotropin-releasing hormone (CRH), a 41-amino-acid peptide, is an important neuromodulator that is widely distributed in the brain [1]. As a neuroendocrine hormone, CRH is abundantly expressed in hypothalamic paraventricular nucleus (PVN) neurons and plays a crucial role in the regulation of the hypothalamic-pituitary-adrenal (HPA) axis [2, 3]. CRH-expressing neurons are also broadly distributed in other brain regions, including the inferior olivary nucleus, Barrington’s nucleus, pontine tegmentum, cerebral cortex, hippocampus, and central amygdala. Depending on their region-specific somatic locations, CRH neurons participate in various functional activities, such as learning memory, synaptic plasticity, food intake, and drug addiction, as well as anxiety-like and depression-like behaviors [4–7].

The anatomy of the brain CRH system has been studied in different mammalian species via immunohistochemistry and radioimmunoassays [8–14]. However, data from these studies have mainly been acquired from histological imaging and through manual reconstruction and counting of labeled neurons, which is time-consuming, limits further systematic analysis, and can introduce biases and/or artifacts. Recently, genetically modified mouse models have been developed to identify the whole-brain distributions of CRH neurons [15–19], which has significantly advanced our understanding of the morphological features of CRH neurons in the rodent brain [20–23]. Advances in whole-brain optical imaging techniques, such as fluorescence micro-optical sectioning tomography (fMOST) [24–27], have made it feasible to further quantify cellular distributions and to morphologically reconstruct cells at the whole-brain level. The precision of imaging via fMOST can reveal complex fiber orientations and can even distinguish individual dendrites. Such quantitative three-dimensional (3D) neuronal morphologies obtained at a brain-wide scale can provide highly accurate arborization details and comprehensive mapping of CRH neuronal connections throughout the brain.

Although the whole-brain expression patterns of CRH have been qualitatively analyzed [17, 18, 28], high-resolution reconstruction of the full morphologies (including both somata and dendrites) of CRH neurons at the single-neuron level has rarely been performed. Since neuronal morphology is considered to be one of the most defining features to distinguish among neuronal types and network connectivities, characterizing single-neuron morphologies may provide key information on how neuronal information and signals are transmitted within the local networks. Furthermore, analysis of detailed morphological information (including data sets of somatic locations, as well as dendritic and axonal morphological features) of diverse CRH neurons may facilitate a better classification of CRH neuronal types and help to reveal their local connectivity. For example, a recent study employed fMOST to investigate CRH distributions in the mouse brain, enabling quantitative analysis of whole-brain CRH somata [18] that has provided us with substantial quantitative information on brain CRH networks. However, at present, the morphological details of individual CRH neuronal fibers at the whole-brain level remain poorly understood. In the present study, we constructed a comprehensive whole-brain map of genetically labeled CRH neurons in the mouse brain, which provides dendritic distribution patterns of single CRH neurons. Reconstructions and further analysis showed that heterogeneous CRH interneurons in the mPFC form layer-dependent dendritic-dendritic and dendritic-somatic connections; furthermore, there was a target-oriented distribution of varicosities within the dendrites of hypothalamic CRH neurons. This work provides a comprehensive description of the whole-brain CRH neuronal distribution pattern and, more importantly, dendritic morphological features of CRH neurons in the mouse brain.

## Results

### Comparison of morphological features of fluorescent-labeled CRH neurons in three fluorescent-reporter mouse lines

**Fig. 1:**
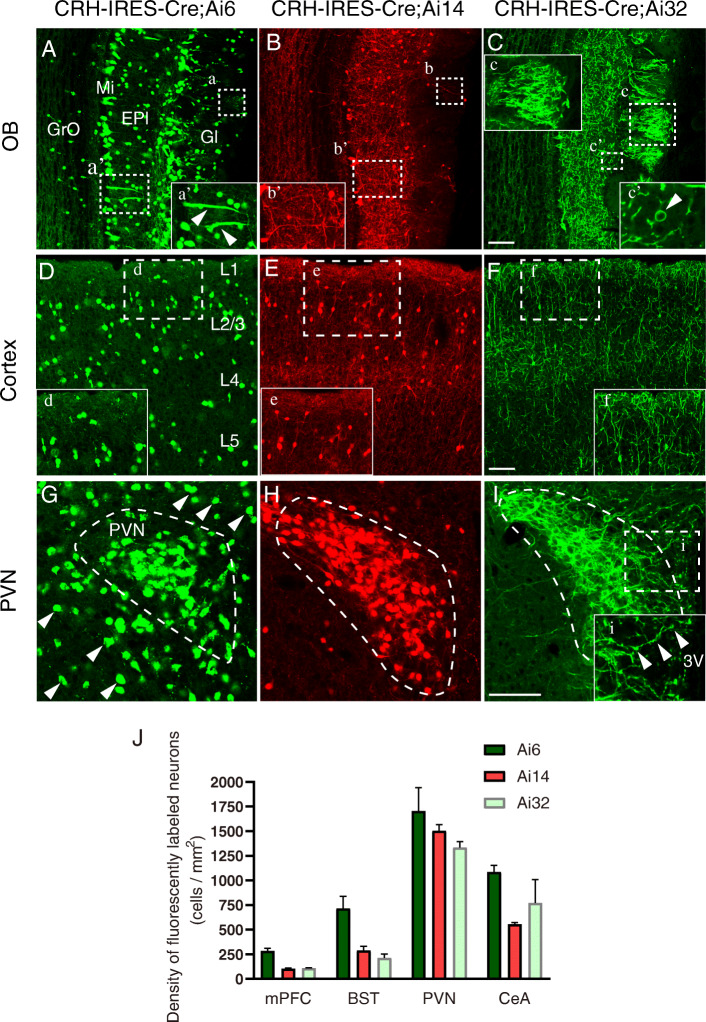
Comparison of the morphological features of CRH neurons in three fluorescent-reporter mouse lines. Comparisons of the distributions and morphologies of fluorescent-labeled CRH neurons in several brain regions of CRH-IRES-Cre;Ai6, CRH-IRES-Cre;Ai14, and CRH-IRES-Cre;Ai32 mice in the OB (**A**–**C**), cortex (**D**–**F**), and PVN (**G**–**I**). (**A**–**C**, **a**–**c**) The dotted boxes show ZsGreen1 (**a**), td-Tomato (**b**), and EYFP (**c**) labeling of synaptic globular structures in the Gl of the three mouse lines. **A**–**B** (**a’**–**b’**) The dotted boxes and magnified images show ZsGreen1 (**a’**) and td-Tomato (**b’**) labeling of cell bodies and dendrites (indicated by the arrowheads) in the Mi and EPl of CRH-IRES-Cre;Ai6 and CRH-IRES-Cre;Ai14 mice. **C** (**c’**) The dotted box and magnified image show EYFP-labeled cell body (indicated by the arrowhead) and dendrites in a CRH-IRES-Cre;Ai32 mouse. **D**–**F** (**d**–**f**) The dotted boxes and magnified images show ZsGreen1 (**d**), td-Tomato (**e**), and EYFP (**f**) labeling of cell bodies and dendrites in layer 1 and layer 2/3 of the three mouse lines. **G** ZsGreen1-labeled cell bodies in (dotted-curved box) and outside (arrowheads) of the PVN of CRH-IRES-Cre;Ai6 mice. **H** Td-Tomato-labeled cell bodies in the PVN (dotted-curved box) of CRH-IRES-Cre;Ai14 mice. **I** (**i**) EYFP-labeled cells (**I**, dotted-curved box) and fibers (**i**, dotted box and magnified image) in the PVN of CRH-IRES-Cre;Ai32 mice. Scale bars = 100 μm. **J** The difference in density of fluorescently labeled neurons in several brain regions of three reporter mouse lines. Data are shown as mean ± SEM; *n* = 4 mice from independent experiments, 4 slices per mouse

By crossing CRH-IRES-Cre mice with Ai6, Ai14, and Ai32 reporter mice, in which the cassette containing ZsGreen1, td-Tomato, or CHR2-EYFP was expressed in a Cre-dependent manner, we obtained CRH-IRES-Cre;Ai6, CRH-IRES-Cre;Ai14, and CRH-IRES-Cre;Ai32 mice, respectively (Additional file 1*,* Figure S1. A). Then, we compared the distributions and morphologies of fluorescent-labeled CRH neurons in several brain regions, including the olfactory bulb (OB) (Fig. 1A, C), cortex (Fig. 1D, F), PVN (Fig. 1G, I), bed nucleus of the stria terminalis (BST) (Additional file 1*,* Figure S1. B and D), and central nucleus of the amygdala (CeA) (Additional file 1*,* Figure S1. E and G).

In the OB, the transgenic fluorescent proteins were mainly distributed in the glomerular layer (Gl), external plexiform layer (EPl), and the mitral cell layer (Mi) in all three mouse lines. CRH-IRES-Cre;Ai6 mice showed the brightest and largest number of fluorescent-labeled cells in all these layers (Fig. 1A); in particular, more fluorescent cells were labeled in the granule cell layer (GrO) in this mouse line compared to those in CRH-IRES-Cre;Ai14 and CRH-IRES-Cre;Ai32 mice. However, fluorescent-labeled neuronal fibers were short and their fluorescent distributions were not uniform; for example, the dendrites close to the cell bodies of mitral cells were strongly labeled (Fig. 1A, a’, indicated by the arrowheads), but the branches extending to the Gl were not clear (Fig. 1A, a, indicated by the dotted box). CRH-IRES-Cre;Ai14 mice were also labeled with bright cell bodies and dense fibers were labeled in the EPl (Fig. 1B, b’), but only a few dendritic structures were labeled in the Gl (Fig. 1B, b, indicated by the dotted box). By contrast, in each layer of CRH-IRES-Cre;Ai32 mice, the fluorescence distributed in cell bodies and fibers exhibited a uniform brightness (Fig. 1C), and the somata in these sections were organized in a ring-like structure (Fig. 1C, c’, indicated by the arrowhead). Unlike the former two mouse lines, the Gl showed a bushy spherical structure (Fig. 1C, c) that was comprised of mitral cells and/or peribulbar cells.

In the cortex, fluorescent-labeled cells were found in each layer in CRH-IRES-Cre;Ai6 mice (Fig. 1D). The cell bodies were strongly labeled, while fibers were rarely seen (Fig. 1D, d). The numbers of labeled cells in the medial prefrontal cortex (mPFC) of CRH-IRES-Cre;Ai14 (103.4 ± 7.9/mm^2^) and CRH-IRES-Cre;Ai32 (108.7 ± 5.1/mm^2^) mice were less than those in CRH-IRES-Cre;Ai6 (283.3 ± 27.8/mm^2^) mice (Fig. 1J, one-way ANOVA, *P* < 0.0001, *F* (2, 9) = 36.56), and the cells were mainly distributed in layer 2/3 (Fig. 1E, F). Neurons in CRH-IRES-Cre;Ai14 mice also showed clearer and brighter cell bodies (Fig. 1E, e), whereas more fibers were labeled (Fig. 1F) in CRH-IRES-Cre;Ai32 mice, especially in terms of a dense distribution in the first layer (Fig. 1F, f).

The outlines of nuclei were clearly visible in the fluorescent labeling of CRH neurons in the PVN (Fig. 1G, I, indicated by the dotted line), BST (Additional file 1*,* Figure S1. B and D, indicated by the dotted box), and CeA (Additional file 1*,* Figure S1. E and G, indicated by the dotted box) of the three mouse lines. Similarly, CRH-IRES-Cre;Ai6 mice showed the highest number of labeled neurons in the PVN (1702 ± 238.9/mm^2^, one-way ANOVA, *P* = 0.2606, *F* (2, 9) = 1.57), BST (714.6 ± 125.3/mm^2^, one-way ANOVA, *P* < 0.005, *F* (2, 9) = 11.38), and CeA (1085 ± 67.35/mm^2^, one-way ANOVA, *P* = 0.0778, *F* (2, 9) = 3.44) (Fig. 1J), as well as the strongest fluorescent labeling within these cell bodies (Fig. 1G; Additional file 1*,* Figure S1. B, a and E, d). In CRH-IRES-Cre;Ai14 mice, distinguishable cell bodies and dense fibers were labeled in the BST (Additional file 1*,* Figure S1. C, b) and CeA (Additional file 1*,* Figure S1. F, e), while only the cell bodies were clearly seen in the PVN (Fig. 1H, indicated by the dotted line). By contrast, CRH-IRES-Cre;Ai32 mice showed dense fibers in all of these regions, and the fluorescent signals of the cell bodies were distinguishable (Fig. 1I, Additional file 1*,* Figure S1. D, c and G, f); especially in the PVN, neuronal fibers extending to the lateral and third ventricle (Fig. 1I, i, fibers indicated by the arrowheads) were visible, and there was a uniform fluorescent intensity distributed in the nearby cell bodies and fibers.

In summary, among the three reporter mouse lines, CRH-IRES-Cre;Ai6 and CRH-IRES-Cre;Ai14 mice showed clearer and brighter cell bodies of CRH neurons. CRH-IRES-Cre;Ai6 mice had the largest number of labeled CRH cells in each tested brain region, but almost no neuronal fibers were visible. CRH-IRES-Cre;Ai14 mice showed clear but incomplete fibers. Only CRH-IRES-Cre;Ai32 mice showed the most complete fibrous structures, especially in terms of distributions in neuronal terminals (e.g., the bushy spherical structures in the glomerular layer of the OB; the extended fibers in the cortex and PVN); regardless of their weaknesses in distinguishing single-cell bodies, the fluorescent distributions in the whole cell were uniform, which is conducive to the adjustment of exposure and the collection of complete morphologies of neurons during imaging.

### Whole-brain distributions of CRH neurons at high resolution in the CRH-IRES-Cre;Ai32 mouse line

**Fig. 2:**
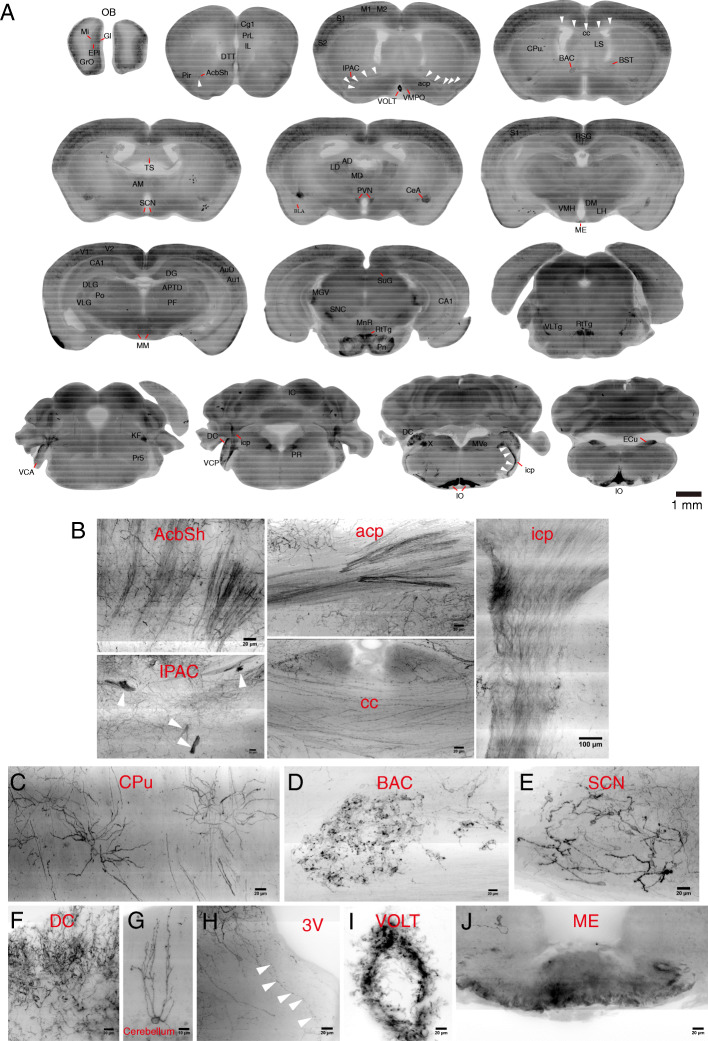
Whole-brain distributions of CRH neurons at high resolution in the CRH-IRES-Cre;Ai32 mouse line. **a** Whole-brain distributions of EYFP-labeled CRH neurons, using the fMOST system, contained in 100-μm coronal-projected sections showing the general distributions of EYFP-labeled CRH neurons in different brain regions. **b** EYFP-labeled clusters of fiber projections from CRH neurons in the AcbSh, IPAC, acp, cc, and icp. **c** EYFP-labeled single neurons in the CPu. **d** Clustered EYFP-labeled neurons distributed in the BAC. **e** EYFP-labeled neurons in the SCN. **f** Dense EYFP-labeled dendrites from neurons in the DC. **g** EYFP-labeled single neuron in the cerebellum. **h** EYFP-labeled fibers and swollen structures around the 3V. **i** EYFP-labeled vascular-like structures in the VOLT. **j** EYFP-labeled terminals of PVN CRH neurons in the ME

Since the single-cell morphology of CRH neurons was most clearly visible in CRH-IRES-Cre;Ai32 mice, we used this mouse line to image EYFP-labeled CRH neurons throughout the brain at a resolution of 0.2 × 0.2 × 1.0 μm via an fMOST system. First, 100-μm down-sampled coronal projection sections (Fig. 2a) were provided to show the overall distributions of CRH neurons in various brain regions. EYFP-labeled cells were distributed in many regions that have not previously been reported, such as in vascular organ of the lamina terminalis (VOLT), ventromedial preoptic nucleus (VMPO), caudate putamen (CPu), bed nucleus of the anterior commissure (BAC), triangular septal nucleus (TS), suprachiasmatic nucleus (SCN), Kölliker-Fuse nucleus (KF), and nucleus X (X) (Fig. 2a). We analyzed the co-localization of EYFP expression with CRH immunoreactivity in these brain regions (Additional file 1*,* Figure S2. A) and found that most of the EYFP-labeled “novel” CRH neurons coexisted with CRH-immunoreactive cells in all of the above brain regions (Additional file 1*,* Figure S2. A, indicated by arrows), but low ratio of co-labeling was observed in some regions such as in the BAC, CPu, SCN, and TS, and some CRH-immunoreactive neurons did not express EYFP. Furthermore, bundles of CRH projection fibers were visible in accumbens nucleus, shell (AcbSh), interstitial nucleus of the posterior limb of the anterior commissure (IPAC), anterior commissure, posterior (acp), corpus callosum (cc), and inferior cerebellar peduncle (icp) (Fig. 2a, indicated by arrowheads and Fig. 2b). The movies of serial sections showed that the fibers in the AcbSh, IPAC, and acp were projections from neurons in the OB (Additional file 2, Movie 1) and that fibers in the icp were projections from IO CRH neurons (Additional file 1*,* Figure S2. B and Additional file 3*,* Movie 2). Moreover, we found novel populations of CRH-positive neurons in some brain regions, such as a sparsely distributed group in the CPu (Fig. 2c) that had dendrites that were radially distributed (with the maximum radius from the terminals to the somata being 40–70 μm). Neurons gathered in the BAC (Fig. 2d) had round cell bodies and two short processes. The average number of CRH-positive neurons found in the SCN was 45.67 ± 0.88, and nearly every neuron had two thick primary dendrites with few branches (Fig. 2e). Neurons in the dorsal cochlear nucleus (DC) (Fig. 2f) had dense apical dendrites distributed in the superficial glial zone, and chandelier cells with apical dendrites vertically distributed were labeled in the cerebellum (Fig. 2g). A cluster of swollen structures (Fig. 2h, indicated by arrowheads) presenting transparent smooth surfaces was visible around the third ventricle (3 V) and always extended to the 3 V border. Densely labeled vascular-like structures and terminals of CRH neurons were found in VOLT (Fig. 2i) and ME (Fig. 2j). The distributions and morphologies of CRH neurons in other brain regions are shown in Additional file 1*,* Figure S2. B.

### Three-dimensional distributions and single-cell reconstructions of CRH neurons in several brain regions

**Fig. 3:**
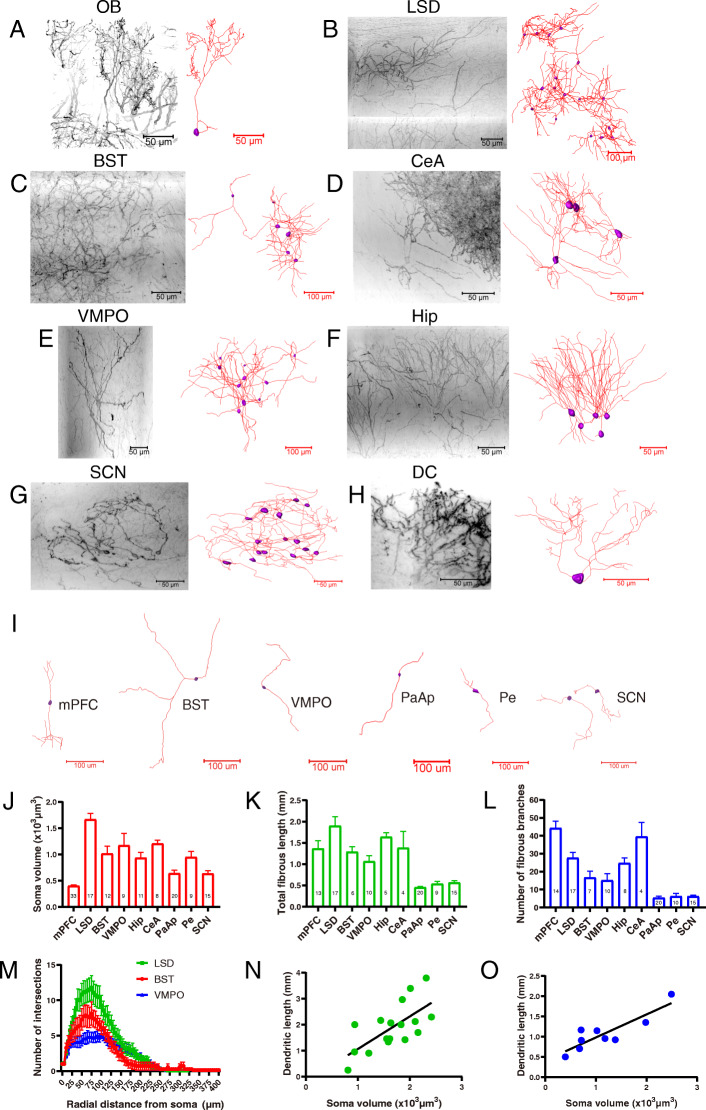
Three-dimensional distributions and single-cell reconstructions of CRH neurons in several brain regions. **a–h** Original images (left half) and reconstructions (right half) of CRH neurons in different brain regions, including the OB (**a**), LSD (**b**), BST (**c**), CeA (**d**), VMPO (**e**), Hip (**f**), SCN (**g**), and DC (**h**). The original images were inverted into grayscale images. The reconstructed somata and dendrites of neurons are indicated by purple bodies and red lines, respectively. **i** Typical reconstructed neurons show the common bipolar morphology found in different brain regions, including the mPFC, BST, VMPO, PaAp, Pe, and SCN. **j** Somatic volumes of CRH neurons in different brain regions [one-way ANOVA, *P* < 0.0001, *F* (8, 125) = 23.24], number in the bars indicate the number of neurons calculated. **k** Total fibrous lengths of CRH neurons in different brain regions [one-way ANOVA, *P* < 0.0001, *F* (8, 90) = 13.93]. **l** Numbers of fibrous branches of CRH neurons in different brain regions [one-way ANOVA, *P* < 0.0001, *F* (8, 96) = 26.94]. **m** Sholl analysis of dendrites of neurons in the LSD, BST, and VMPO illustrate changes in the mean number of intersections with increasing radial distance from the soma, *n* = 16 cells for LSD, 7 cells for BST and 10 cells for VMPO. **n** Correlation between dendritic length and somatic volume in LSD neurons (Pearson’s correlation coefficient *r* = 0.68, *P* = 0.0029). **o** Correlation between dendritic length and somatic volume in VMPO neurons (Pearson’s correlation coefficient *r* = 0.88, *P* = 0.0019)

We reconstructed EYFP-labeled CRH neurons in several brain regions (Fig. 3a, h), including the OB (Fig. 3a), dorsal part of lateral septal nucleus (LSD) (Fig. 3b), BST (Fig. 3c), CeA (Fig. 3d), VMPO (Fig. 3e), hippocampus (Hip) (Fig. 3f), SCN (Fig. 3g), and DC (Fig. 3h). We found that the reconstructed neurons in several brain regions (e.g., mPFC, BST, VMPO, anterior parvicellular part of paraventricular hypothalamic nucleus (PaAp), periventricular hypothalamic nucleus (Pe), and SCN) shared similar morphological characteristics consistent with bipolar neurons (Fig. 3i). CRH neurons in the LSD had the largest average volume of somata (1632 ± 159.6 μm^3^) (Fig. 3j) and the longest dendritic length (1.9 ± 0.2 mm) (Fig. 3k). Dendritic length significantly increased as a function of somatic volume (*R*^2^ = 0.597, *P* = 0.0032) (Fig. 3n). CRH neurons in the VMPO also had a larger cell bodies (1172 ± 228.1 μm^3^) (Fig. 3j), but the number of dendritic branches (15.2 ± 3.7) (Fig. 3l) and dendritic length (1.1 ± 0.1 mm) (Fig. 3k) was less than those of neurons in the LSD. Dendritic length was also positively correlated with somatic volume in the VMPO (Fig. 3o). Sholl analysis showed that neurons in the LSD had the largest maximum number of intersections, while the VMPO had the least maximum number of intersections. For all of these regions, the maximum numbers of intersections were located at radial distances of 50–100 μm from the somata (Fig. 3m). The more complex dendrites of CRH neurons in the LSD, compared to those in other areas, suggested that CRH neurons in the LSD may receive comparatively more inputs. Most of the dendritic morphologies of CRH neurons in the hippocampus exhibited a similar pattern of an umbrella shape of upward dendrites (Fig. 3f). CRH neurons in the SCN were scattered throughout the nucleus and the dendrites were interlaced with one another (Fig. 3g). We next compared the parameters of all reconstructed neurons (Additional file 1*,* Table S1) in different brain regions and found that CRH neurons in hypothalamic regions—including the PaAp (640.1 ± 60.4 μm^3^), Pe (951.2 ± 108.3 μm^3^), and SCN (636.0 ± 55.4 μm^3^)—had smaller somatic volumes (Fig. 3j and Additional file 1*,* Table S1). Similarly, there were also shorter dendritic lengths of CRH neurons in the PaAp (0.5 ± 0.02 mm), Pe (0.5 ± 0.06 mm), and SCN (0.6 ± 0.05 mm) (Fig. 3k and Additional file 1*,* Table S1). The simpler morphologies of hypothalamic CRH neurons may be related to their endocrine and other conserved functions.

### Multiple morphological types of CRH neurons form distinct dendritic connections in the medial prefrontal cortex (mPFC)

**Fig. 4:**
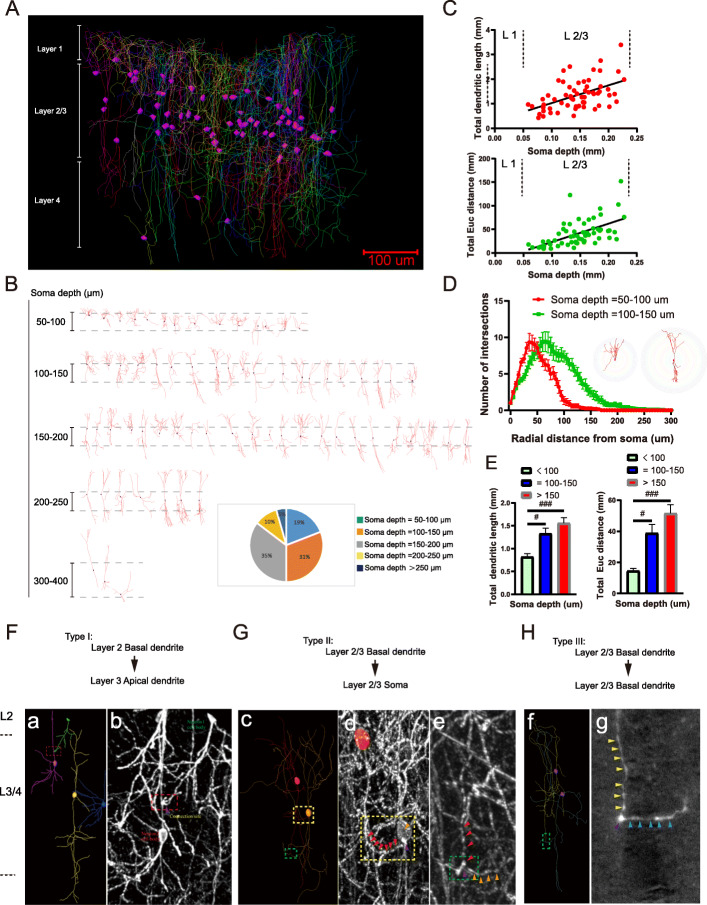
Multiple morphological types of CRH neurons form putative connections in the mPFC. **A** Overview of reconstructed CRH neurons including somata (indicated by the purple bodies) and fibers (indicated by the colored lines) from layers 1–4 in a column with a volume of 350 × 500 × 500 μm in a PrL subregion within the mPFC. **B** Representation of 67 reconstructed somata and dendrites of CRH neurons arranged along their somatic depths with respect to the pial surface; the pie chart at the bottom right shows the percentages of neurons distributed at different somatic depths. **C** Upper half: Correlation between total dendritic length and somatic depth in PrL neurons (Pearson’s correlation coefficient *r* = 0.51, *P* < 0.0001); Lower half: Correlation between total Euclidean distance and somatic depth in PrL neurons (Pearson’s correlation coefficient *r* = 0.59, *P* < 0.0001). **D** Sholl analysis of the dendrites of neurons with somatic depths = 50–100 μm and 100–150 μm illustrating changes in the mean number of intersections with increasing radial distance from the soma. Inset images show exemplary intersections on two typical neurons (left: somatic depth = 50–100 μm, right: somatic depth = 100–150 μm) with different somatic depths, *n* = 15 cells for somatic depths = 50–100 μm and 19 cells for somatic depths = 100–150 μm. **E** Left: The total dendritic length of CRH neurons with somatic depths < 100 μm showed differences compared with those with somatic depths = 100–150 μm (one-way ANOVA, multiple comparisons, *P* = 0.0285) and soma depths > 150 μm (one-way ANOVA, multiple comparisons, *P* = 0.0005). Right: The total Euclidean distance of CRH neurons with a somatic depth of < 100 μm showed differences compared with those with soma depths = 100–150 μm (one-way ANOVA, multiple comparisons, *P* = 0.0213) and somatic depths > 150 μm (one-way ANOVA, multiple comparisons, *P* = 0.0001), *n* = 13 cells for somatic depths < 100 μm, 21 cells for 100–150 μm and 31 cells for > 150 μm. **F**–**H** Three types of connections between CRH neurons in the mPFC. **F** **a** (reconstructed neurons) and **b** (original image) show the type-I connection (the connection site is indicated by the purple arrowhead in the red dotted box). **G** The yellow-dotted boxes in **c** (reconstructed neurons) and **d** (original image) show the type-II connection (red arrowheads indicate a branch of basal dendrites of one neuron and the orange arrowhead indicates the cell body of another neuron; the connection site is indicated by the purple arrowhead). Green-dotted squares in **c** (reconstructed neurons) and **e** (original image) showed the type-III connection (red arrowheads indicated a branch of basal dendrites of one neuron and the orange arrowheads indicate a branch of basal dendrites of another neuron; the connection site is indicated by the purple arrowhead). **H** Green-dotted squares in **f** (reconstructed neurons) and **g** (original image) also showed the type-III connection (yellow arrowheads indicate a branch of basal dendrites of one neuron and the blue arrowheads indicate a branch of basal dendrites of another neuron; the connection site is indicated by the purple arrowhead)

Recent studies have shown that CRH neurons in the mPFC play a critical role in higher cognitive functions [29, 30]. In the prelimbic cortex (PrL) within the mPFC, we reconstructed the entire somata (Fig. 4A, purple bodies) and dendrites (Fig. 4A, color lines) of EYFP-labeled CRH neurons within a column that had a volume of 350 × 500 × 500 μm. The cell bodies of these neurons were mostly distributed within layers 2–4 and most of their dendrites were vertically distributed. There were dendritic branches in both the upper and lower parts of the somata. The apical dendrites that branched in the first layer formed a dense dendritic network, and most of them reached the pia mater (Fig. 4A, reconstructed fibers indicated in layer 1); furthermore, the basal dendrites extended and branched into layer 4 at a distance of approximately 500 μm from the cortical surface (Fig. 4A, reconstructed fibers indicated in layer 4). Individual reconstructed neurons were classified according to the distances (50–100, 100–150, 150–200, 200–250, and > 250 μm) between their somata and the surface of the cortex (Fig. 4B), and the percentages of neurons in these categories were 19%, 31%, 35%, 10%, and 5%, respectively (Fig. 4B, indicated in the pie chart). We found that there was significant correlation between the somatic depth (distance from the cortical surface) and both the total dendritic length (Fig. 4C, upper half, *r* = 0.4440) and total Euclidean distance (the straight-line distance from the soma to the given point of the dendrite) (Fig. 4C, bottom half, *r* = 0.5399). Sholl analyses showed that the number of intersections with a radial distance from the soma being less than 50 μm was larger in neurons with a somatic depth of 50–100 μm than that of neurons with a somatic depth of 100–150 μm; in contrast, the number of intersections with a radial distance from the soma being more than 50 μm was smaller and ended at approximately 100 μm of the radial distance from the soma in neurons with a somatic depth of 50–100 μm. Interestingly, the maximum numbers of intersections were similar between these two types of neurons (Fig. 4D). Sholl analyses of CRH neurons with different somatic depths are shown in additional file 1*,* Figure S3. J. We also found that the total dendritic length (Fig. 4E, left half, one-way ANOVA, *P* = 0.0008, *F* (2, 60) = 8.100) and the total Euclidean distance (Fig. 4E, right half, one-way ANOVA, *P* = 0.0002, *F* (2, 60) = 9.915) of neurons with a somatic depth of less than 100 μm were significantly smaller than those with somatic depths of 100–150 μm (*P* = 0.0285) and more than 150 μm (*P* = 0.0005), while there were no significant differences in the total number of dendritic branches or the total number of dendritic terminal points (Additional file 1*,* Figure S3, K). The average dendritic lengths of neurons at somatic depths of less than 100 μm, 100–150 μm, and more than 100–150 μm were 0.82 ± 0.26, 1.33 ± 0.54, and 1.48 ± 0.68 mm, respectively; furthermore, their total Euclidean distances were 14.65 ± 5.19, 38.77 ± 25.49, and 48.67 ± 30.38 mm, and their total numbers of dendritic branches were 23.23 ± 8.65, 32.86 ± 20.28, 33.32 ± 19.44, respectively.

To investigate local CRH-CRH connection patterns within the cortex, we divided CRH-CRH connections into three types (Fig. 4F, H). Type I consisted of basal-to-apical connections. Here, somata in layer 2 sent dendrites downward (the green cell of Fig. 4F, a) that contacted with the upward dendrites (the purple cell of Fig. 4F, a) from the somata in layer 3 (as shown in the red dotted box of Fig. 4F, a, b). Type II consisted of basal-to-somatic connections (as shown in the yellow-dotted box of Fig. 4Gc, d). In layer 2–3, a soma in the upper layer sent dendrites downward (as shown in Fig. 4G, c, red cell), and the end of one branch (Fig. 4G, d red arrows) was in contact with an adjacent lower cell body (Fig. 4G, c, orange cell; Fig. 4G, d, orange arrows). Type III consisted of basal-to-basal connections (Fig. 4G, c, e; Fig. 4H, f, green-dotted box). Two cell bodies in layers 2–3 sent dendrites downward, and the end of one branch (Fig. 4G, e, red arrows; Fig. 4H, g, yellow arrows) from the upper soma and the branch (Fig. 4G, e, orange arrows; Fig. 4H, g, blue arrows) from the lower soma formed a connection. A common feature of the three types of connections was that the fluorescent intensity increased at the contact point, indicating a possible connection of structures (Fig. 4F, b, G, d and e, and H, g, purple arrows). Examples of type-II and type-III connections are demonstrated in Additional file 4*,* Movie 3.

We next performed immunofluorescent staining to determine the specificity of EYFP-labeled neurons in CRH-IRES-Cre;Ai32 mice. The results showed that most of the EYFP-labeled neurons in the mPFC were CRH-immunoreactive cells (Additional file 1 Figure S3, A–C, indicated by white arrowheads). We further identified that these CRH interneurons were GAD67-GFP-positive neurons (Additional file 1, Figure S3, D-F, indicated by white arrowheads) by using CRH-IRES-Cre;Ai14;GAD67-GFP mice. Interestingly, in adult mouse brains, EYFP-labeled pyramidal neurons were visible in layer 3 or layer 5 of the cortex (Additional file 1 Figure S3, H), but there were no EYFP-labeled pyramidal neurons on the 21st day after birth (Additional file 1*,* Figure S3*,* G). These fluorescently labeled pyramidal neurons were not CRH-immunoreactive cells (Additional file 1*,* Figure S3*,* I), including within their dendrites and spines (Additional file 1, Figure S3*,* a, indicated by arrowheads). We also observed that some EYFP-labeled neurite swellings in layer 1 were also labeled with CRH antibodies (Additional file 1, Figure S3*,* b, indicated by arrowheads).

### Reconstructions and morphological features of CRH neurons in the PaAp and Pe

**Fig. 5:**
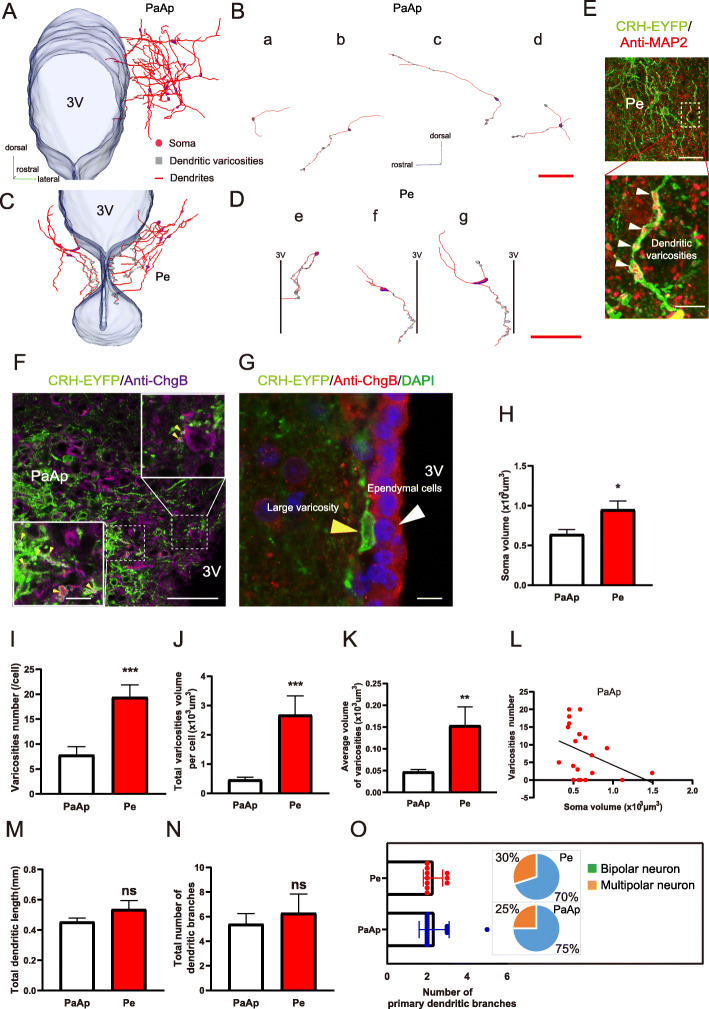
Reconstructions and morphological features of CRH neurons in the PaAp and Pe. **A** Three-dimensional distribution of reconstructed whole morphologies of CRH neurons in the PaAp. The somata are indicated by the purple bodies, the gray bodies indicate the dendritic varicosities, and the red lines indicate the dendrites; the transparent structure on the left represents the reconstructed 3V. **B** Four typical reconstructed neurons in the PaAp. **B** (**a**) A bipolar neuron with short dendrites and no varicosities; **B** (**b**) a bipolar neuron with varicosities located on the dendrite at one end of the cell body. **B** (**c**) A bipolar neuron with varicosities located on dendrites at both ends of the cell body; **B** (**d**) a multipolar neuron with varicosities located on the dendrites (scale bar = 100 μm). **C** Three-dimensional distribution of reconstructed whole morphologies of CRH neurons in the Pe. The somata are indicated by purple bodies, the gray bodies indicate the dendritic varicosities, and the red lines indicate the dendrites; the transparent structure in the center represents the reconstructed 3V. **D** Three individual reconstructed neurons in the Pe. **D** (**e**) A bipolar neuron with both ends of dendrites extending to the 3V and with varicosities located on the dendrites at both ends of the cell body; **D** (**f**) a bipolar neuron with one end of the dendrite extending to the 3V and with varicosities located on the dendrite at one end of the cell body; **D** (**g**) shows a multipolar neuron with one dendrite extending to the 3V and with varicosities mostly located on the dendrite at one end of the cell body (scale bar = 100 μm). **E** EYFP-labeled varicosities contain MAP 2-immunopositive signals (indicated by the arrowheads, image below is a magnified image from the dotted box of the upper image, scale bar: 50 μm and 10 μm for the magnified image). **F** EYFP-labeled varicosities contain ChgB-immunopositive signals (indicated by the arrowheads in the magnified images from the dotted boxes, scale bar: 50 μm and 10 μm for the insert). **G** The dendrite extends to the third ventricle and a large varicosity (indicated by the yellow arrowhead) is attached to the ependymal cells (indicated by the white arrowhead, scale bar: 10 μm). **H**–**K** Statistical results showing the differences of somatic volume (**H**) (*P* = 0.0119), varicosities number (**I**) (*P* = 0.0005), total varicosities volume per cell (**J**) (*P* = 0.0003), and the average volume of the varicosities (**K**) (*P* = 0.0047) between neurons in the PaAp and Pe. **L** Correlation between somatic volume and varicosities number of neurons in the PaAp. Spearman’s correlation coefficient was *r* = − 0.4519, *P* = 0.0455. Each point in the scatterplot represents a single cell. **M**, **N** There were no significant differences in dendritic features (**M**, total dendritic length; **N**, total number of dendritic branches) of neurons between PaAp and Pe. ns: not significant. **O** Comparison of primary dendritic branches and the proportions of bipolar or multipolar cells in the PaAp and Pe. *N* = 20 cells for PaAp and 10 cells for Pe

Hypothalamic neuroendocrine CRH neurons play an important role in stress responses, but neurons within different subregions require more detailed morphological analysis. We chose EYFP-labeled neurons in the PaAP and Pe to reconstruct their somata and processes (Fig. 5A, C; Additional file 1, Figure S4*,* A and B). There was a noteworthy co-localization pattern (Additional file 1*,* Figure S4*,* E–G) for the EYFP-labeled signals and CRH immunoreactivity in the PaAP. There were vesicular fluorescent labels (dendritic varicosities) (Fig. 5B, D, gray reconstructed structures) on the neurites of neurons in both the PaAP and Pe, and they also co-labeled with CRH immunopositive-structures (Additional file 1, Figure S4, H, indicated by arrowheads). In terms of their 3D patterns, the somata of some neurons (Fig. 5A, B, purple reconstructed cell bodies) distributed in the PaAP sent out fibers rostrally (Fig. 5A, B, red lines), and there were spaced and small dendritic varicosities (Fig. 5B, b–d, gray bodies) on these fibers. An example of these reconstructed cells is shown (Fig. 5B) according to the primary branches and number of dendritic varicosities, and there were four distribution patterns of these neurons. Pattern 1 (Fig. 5B, a) consisted of cells that had two primary branches with the shortest dendritic length and no varicosities. Pattern 2 (Fig. 5B, b) consisted of cells that had two primary branches with similar dendritic lengths at both ends of the somata and were distributed almost vertically, and the fibers extending rostrally had varicosities. Pattern 3 (Fig. 5B, c) consisted of cells with both ends of the dendrites having varicosities and the one dendritic branch that was distributed horizontally was longer and extended rostrally, whereas the other short branch was distributed vertically. Finally, pattern 4 (Fig. 5B, d) consisted of dendrites of multipolar cells extending rostrally having varicosities and being distributed horizontally. The locations of the reconstructed somata in the PaAP are shown in Additional file 1, Figure S4 A (purple bodies indicated by red circles).

In the Pe, the reconstructed somata were located in the lower part of the PaAp and around the 3V (Fig. 5C, purple bodies; Additional file 1*,* Figure S4, B, indicated by red circles). These cells sent out fibers and one or two of them extended close to the 3V (Fig. 5C, D, red line). There were large varicosities on the dendrites (Fig. 5D, gray reconstituted bodies; Fig. 5E, indicated by white arrowheads) and they terminated (Fig. 5G, indicated by the yellow arrowhead) near the ependymal cell (Fig. 5G, indicated by the white arrowhead) layer adjacent to the 3V. Most of the reconstructed cells within the Pe were bipolar neurons (Fig. 5D, e, f), and the fibers extending downward to the 3V had more varicosities (Fig. 5D, f, g). We further identified the immunopositive substances contained in these varicosities and found that there were extentive MAP 2 -immunopositive signals (a marker of dendrite) in the varicosities (Fig. 5E, indicated by white arrowheads; Additional file 1*,* Figure S4*,* I). Chromogranin B (ChgB) immunoreactivity (associated with large dense core vesicles) was also found in the dendritic varicosities in PaAp (Fig. 5F) and Pe (Additional file 1*,* Figure S4*,* J-L) indicating that these varicosities contain a large amount of dense core vesicles. Interestingly, the ependymal cells adjacent to the 3V were also found to be ChgB immunopositive (Fig. 5G). The dendritic varicosities were also stained with Kinesins (molecular motors used for intracellular transport and trafficking) in PaAp (Additional file 1*,* Figure S4*,* M-O) and Pe (Additional file 1*,* Figure S4*,* P-R).

Next, we compared the somatic and dendritic parameters of the neurons in the PaAP and Pe and found that the average volume of the soma in the Pe (951.2 ± 108.3 μm^3^) was larger than that in the PaAP (640.1 ± 60.4 μm^3^) (Fig. 5H, *P* = 0.0119, *t* = 2.697), and the number of dendritic varicosities in the Pe (19.4 ± 2.5) was significantly greater than that in the PaAP (7.9 ± 1.6) (Fig. 5I, *P* = 0.0005, *t* = 3.974). The total dendritic varicosities volume (2678 ± 652.3 μm^3^) (Fig. 5J, *P* = 0.0003, *t* = 4.348) per cell and the average volume of dendritic varicosities (153.4 ± 43.1 μm^3^) (Fig. 5K, *P* = 0.0047, *t* = 3.148) in the Pe were significantly larger than those (468.2 ± 79.2 μm^3^ and 47.7 ± 5.3 μm^3^) in the PaAP. We also found that there was a negative correlation between the number of dendritic varicosities and the soma volume in the PaAP (*r* = − 0.4519, *P* = 0.0455) (Fig. 5L). There was no significant difference in the total dendritic length (PaAP: 455.4 ± 24.1 μm, Pe: 537.5 ± 57.6 μm) (Fig. 5M) or the total number of dendritic branches (PaAP: 5.4 ± 0.9, Pe: 6.3 ± 1.6) (Fig. 5N) of neurons in the PaAP and Pe. We found that most (75% in the PaAP and 70% in the Pe) (Fig. 5O, right half, indicated in the pie chart) of the reconstructed neurons were bipolar neurons, which were characterized by the number of primary dendritic branches (Fig. 5O, left half).

Collectively, these 3D reconstructions of hypothalamic CRH neurons may be indicative of the transport and storage of CRH peptides in hypothalamic neurons, as well as the possible their release sites, such as the third ventricle. These findings provide a structural basis for further elucidating the neural circuits and functions of CRH neurons.

### Arborization-dependent dendritic spine characteristics of CRH neurons

**Fig. 6:**
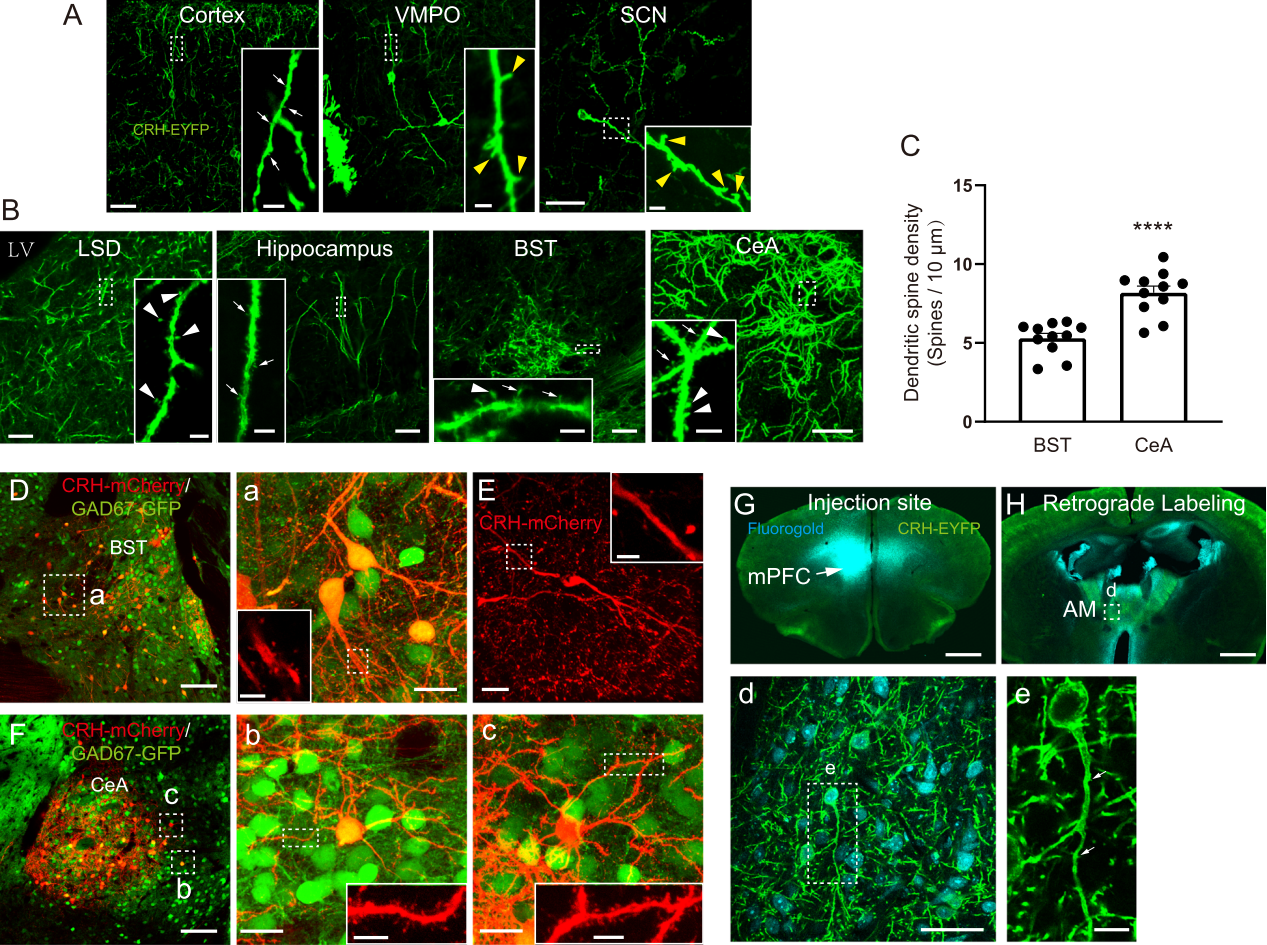
Dendritic spine characteristics of CRH neurons. **A** The dendritic spine characteristics of aspiny CRH neurons in brain regions of the cortex, VMPO, and SCN. The thin spines are indicated by the arrows and the filopodia-like spines are indicated by the yellow arrowheads. **B** The dendritic spine characteristics of spiny CRH neurons in brain regions of the LSD, hippocampus, BST, and CeA. The thin spines are indicated by the arrows and the mushroom-like spines are indicated by the white arrowheads. The inserted images are magnified images from the dotted boxes (scale bar: 50 μm and 5 μm for the inserts). **C** The difference in dendritic spine density between the BST (*n* = 11) and CeA (*n* = 11) (*P* < 0.0001, *t* = 5.467). **D** (**a**) The GAD67-GFP-positive CRH neurons in the BST are spiny. **a** is a magnified image from the dotted box in **D**. **E** The aspiny CRH neurons in the BST. **F** (**b**) and (**c**) The GAD67-GFP-positive (**b**) and -negative (**c**) CRH neurons in the CeA are spiny. **b** and **c** are magnified images from the dotted box in **F**. The inserted images are magnified images from the dotted boxes (scale bar: 50 μm for **D** and **F**, 10 μm for **E** and **a**–**c**. and 5 μm for the inserts). **G**–**H** The retrograde-labeled CRH neurons in the AM (**H**, **d**) by injecting fluorogold in the mPFC (**G**) are aspiny (**e**, the thin spines are indicated by the arrows). **d** and **e** are magnified images (scale bar: 1 mm for **G** and **H**, 50 μm for **d**, and 5 μm for **e**)

We further detected and analyzed the characteristics of dendritic spines of CRH neurons. In general, CRH neurons with sparse dendritic branches had less spines. Consistent with previous studies, mushroom-like and thin dendritic spines were found in the cortex, hippocampus, BST, and CeA (Fig. 6A, B). There were also several areas containing CRH neurons with dendritic spines that have not previously been reported. CRH neurons in the VMPO and SCN were aspiny with few strong filopodia-like spines (Fig. 6A, VMPO and SCN, indicated by arrowheads), the maximum lengths of which reached 5 μm. Furthermore, CRH neurons in the LSD had mushroom-like spines (Fig. 6B, LSD, indicated by arrowheads). CRH neurons with few dendritic branches appeared to be aspiny, such as bipolar CRH neurons in the cortex, VMPO, SCN (Fig. 6A), and BST (Fig. 6E), while CRH neurons with many branches (CRH neurons showed in Fig. 6B) were spiny. In the BST and CeA, we further calculated the densities of dendritic spines and found that CRH neurons in the CeA had more spines than those in the BST (Fig. 6C, *P* < 0.0001, *t* = 5.467, 11 different lengths of dendrites from three mice were calculated). Most oGAD67-GFP-positive CRH neurons in both the BST (Fig. 6D, a) and CeA (Fig. 6F, b) were spiny, while aspiny CRH neurons (Fig. 6E) and GAD67-GFP-negative spiny CRH neurons (Fig. 6c) were also found in the BST and CeA. Interestingly, by injecting fluorogold into the mPFC (Fig. 6G) in CRH-IRES-Cre;Ai32 mice, we found that long-range-projecting CRH neurons that were co-labeled with fluorogold (Fig. 6H, d) in the anteromedial thalamic nucleus were aspiny (Fig. 6e, thin spines indicated by the arrows).

## Discussion

During the last few decades, transgenic rodent models have become powerful tools for studying the distribution [15, 17, 19] and function [31–34] of CRH neurons in the brain. In order to probe the morphological characteristics of CRH neurons at single-cell resolution, we combined genetic labeling (using transgenic mouse lines) with the fMOST platform to generate high-resolution imaging datasets, with which we characterized the morphologies of distinct CRH neurons distributed in various brain regions throughout the whole mouse brain.

The robust native fluorescence of each of these reporter mouse lines enabled direct visualization of fine dendritic and axonal structures of labeled neurons, which has been demonstrated to be useful for mapping neuronal circuitry, as well as imaging and tracking specific cell populations [35–37]. We compared the distribution patterns of fluorescent-labeled CRH neurons in three reporter mouse lines. We found that adult CRH-IRES-Cre;Ai6 mice showed the highest number of labeled neurons in several brain regions (Fig. 1J). Although the three mouse lines were designed in a similar manner, the results may have been due to the sensitivity to Cre and strength of fluorescent reporters. Ai6 reporter lines are more sensitive to low levels of Cre, leading to a more thorough identification of Cre-positive populations [35], and the expression of the enhanced fluorescent protein ZsGreen1 were more easily to be seen. Another possible explanation is that Cre-mediated recombination had occurred in more cells in Ai14 or Ai32 reporter lines, but it was undetected owing to low reporter expression. Notably, the fluorescent fusion protein, CHR2-EYFP, is membrane-bound and is therefore distributed along the plasma membrane of neuronal processes within CRH-IRES-Cre;Ai32 mice [38, 39], which enables a clear visualization of the entire neuronal morphology. Therefore, we utilized CRH-IRES-Cre;Ai32 mice for whole-brain imaging and reconstructions. Interestingly, a large number of EYFP-labeled cortical pyramidal neurons was also observed in adult mice (which has not been reported previously from the onset age of postnatal day 21) (Additional file 1*,* Figure S3).

Next, we focused our analyses of reconstructed neurons mainly in several stress-related regions, including the mPFC, hypothalamus, amygdala, BST, and hippocampus. For example, it has been reported that local CRH-synthesizing neurons are prominent in the PFC [8, 40–43] and may modulate the activities of pyramidal neurons [7]. However, until now, the complete morphologies of CRH neurons in the mPFC have rarely been reported. Here, we reconstructed fluorescent-labeled CRH neurons in the cortical column in the PrL within mPFC across layers 1–4 (Fig. 4A). Importantly, we classified different neuronal types by their soma depths and arborization patterns (example listed in Fig. 4B). For the first time, we showed the distribution of CRH neurons with different morphological types in different cortical layers. We found there were dense dendrites (Fig. 4A) with dendritic swellings (Additional file 1*,* Figure S3, b) in layer 1 (the soma of which were located in layer 2/3 or layer 4), and that fibers extended to the surface of the cortex. According to the layer-specific dendritic locations and their different projection targets, several types of putative connection patterns between CRH neurons were identified in the cortex (listed in Fig. 4F–H). Such a diverse dendritic connection pattern of cortical CRH neurons may reflect differential innervation of downstream output targets (each amplified subfigure shown in Fig. 4F–H). Therefore, by characterizing their somatic locations and unique respective local dendritic morphologies of CRH neurons, our present study not only increases our current understanding of the distribution of CRH neurons, but also enables future studies to further elaborate upon cell-specific classifications. Taken together, these findings may help elaborate future functional studies of morphologically diverse CRH neurons in the PFC.

Importantly, CRH functions as a neuropeptide hormone produced in neuroendocrine neurons in the PVN and regulates the synthesis and secretion of glucocorticoids from the adrenal glands through the action of adrenocorticotropic hormone. For the first time, we reconstructed the intact morphologies of CRH neurons and their neurite varicosities located within dendrites (Fig. 5E) in the PaAp (Fig. 5A, B) and Pe CRH neurons (Fig. 5C, D). In the PaAp, most dendrites with varicosities projected rostrally (Fig. 5B), while in the Pe, the ends of the dendrites extended to the third ventricle and the large varicosities were attached to ependymal cells (Fig. 5C–D, G). We further identified that these dendritic varicosities contained the large dense core vesicle-associated protein, ChgB, and molecular motors (e.g., kinesins) used for intracellular transport and trafficking. Interestingly, a number of varicosities at the end of a dendrite located closely to the out layer of ependymal cells to third ventricle which were ChgB immunopositive (Fig. 5G). In addition, there were no EYFP-labeled CRH fibers distributed in the ependymal cells or passed through the cells. These results suggested that fibers of CRH neurons in the Pe make direct contacts to ependymal cells and may release to the 3rd ventricle by ependymal cells. Therefore, we speculate that these endocrine CRH neurons are different from those that project to the median eminence and that CRH may be also released by dendrites to other areas of the hypothalamus or cerebrospinal fluid to participate in its regulatory functions. We further found a negative correlation between somatic volume and varicosities number in the PaAp (Fig. 5L). Thus, our reconstructed morphological characteristics of dendritic varicosities may facilitate future classifications (according to different fiber orientations and varicosities distribution patterns) of hypothalamic CRH neurons and advance our understanding of their potentially diverse functions.

The reconstructed CRH neurons in different brain regions showed diverse distribution patterns and morphologies (Fig. 3a–h). We found that some neurons shared a common bipolar shape across various brain regions (Fig. 3i), especially in the hypothalamus (75% in the PaAp, 100% in SCN) and cortex. It has been reported that parvocellular CRH neuroendocrine neurons typically have two relatively thick primary dendrites that extend from opposite sides of the soma in a bipolar arrangement and branch once [44, 45]; furthermore, bipolar cells are commonly found in the cortex [12, 46, 47]. Thus, our present study in CRH-reporter mice is consistent with these previous studies and is the first to describe the specific neural structures of these CRH neurons, such as the different types of connections between CRH neurons in the mPFC, as well as the intact morphologies of dendritic varicosities in hypothalamic CRH neurons. Such simplified branching properties of these CRH neurons in the hypothalamus may be conducive to their endocrine functions. Among all the reconstructed CRH neurons across different brain regions, their somata had different sizes (Fig. 3j), with somata in the LSD, VMPO, and hippocampus being larger than those in the PaAp, Pe, and SCN. Also, we found differential dendritic branch complexities across these regions. For example, CRH neurons in the mPFC, LSD, and hippocampus all exhibited more complex dendritic morphologies compared to those in the PaAP, Pe, and SCN (Fig. 3k–l). Hupalo et al. demonstrated that chemogenetic activation of caudal but not rostral dmPFC CRH neurons potently impaired working memory, whereas inhibition of these neurons improved working memory [29]. In addition, CRH acts in the medial septum to impair spatial memory [48] and acts in the BNST to participate in stress-induced maladaptive behaviors [49]. However, the functions of CRH in the OB, SCN, and VMPO remain unclear. Therefore, our current study may provide a detailed morphological basis for future functional-based studies on CRH neurons in these different brain regions. Interestingly, the PaAP, Pe, and SCN are all contained within the hypothalamus, and CRH neurons in these regions had smaller somata and exhibited more prevalent bipolar branching patterns compared to those of other brain regions analyzed in our present study. In the PVN, CRH neurons have been identified as parvocellular cells [44, 50]. We found that the mean somatic volume of CRH neurons in the Pe was larger than that of CRH neurons in the PaAP (Fig. 5H). Hence, we speculate that these data may be indicative of two different types of CRH endocrine neurons within the hypothalamus. Collectively, our quantitative analysis of these reconstructions demonstrates a region-specific diversity of CRH neurons in terms of both somatic size and branching complexity.

Dendritic spines are conventionally believed to be largely absent from inhibitory neurons. Previous studies by other groups and our previous research have shown that CRH neurons are GABAergic neurons that are located in many different brain regions, such as in the cortex [30] and hippocampus; furthermore, CRH neurons are usually aspiny, while some long-range projecting CRH neurons in the BST and CeA have been reported to have spines [38]. In our present study, the arborization-dependent pattern of dendritic spines of CRH neurons was detected where the most sophisticated types of spines in the extended amygdala (BST and CeA) and the simplest one in the hypothalamus (VMPO and SCN). Interestingly, while spiny GABAergic CRH neurons in the BST and CeA were confirmed, aspiny CRH neurons were also found in these areas.

## Conclusions

In summary, in the present study, we generated high-resolution imaging datasets to characterize, at single-cell resolution, the fine morphologies of CRH neurons distributed in diverse brain regions. Such region-specific reconstructions of intact morphologies of CRH neurons may help in further elucidating both CRH-mediated physiological functions in various brain circuits and the associations of their dysfunction in various neuropathological diseases.

## Methods

### Animals

CRH-IRES-Cre (B6(Cg)-Crh^tm1(cre)Zjh^/J; stock number: 012704), Ai6 (B6.Cg-Gt (ROSA)26Sor^tm6(CAG-ZsGreen1)Hze^/J; stock number: 007906), Ai14 (B6.Cg-Gt (ROSA)26Sor^tm14(CAG-TdTomato)Hze^/J; stock number: 007914), Gad67-GFP, and Ai32 (B6;Cg-Gt (ROSA)26Sor^tm32(CAG-COP4*H134R/EYFP)Hze^/J; stock number: 012569) mice have been described previously [35, 36, 51, 52]. CRH-IRES-Cre, Ai6, and Ai32 mice were purchased from Jackson Laboratory. Gad67-GFP and Ai14 mice were obtained from the laboratories of Fuqiang Xu (WIPM, China) and Minmin Luo (NIBS, China), respectively. All of the mice were bred onto a C57BL/6 J genetic background. CRH-IRES-Cre;Ai6, CRH-IRES-Cre;Ai14, and CRH-IRES-Cre;Ai32 mice were derived from crosses of CRH-IRES-Cre/Ai6, Ai14, and Ai32 genotypes, respectively. CRH-IRES-Cre;Ai14;Gad67-GFP mice were derived from crosses of CRH-IRES-Cre;Ai14 and Gad67-GFP genotypes. Male mice at 8–12 weeks of age were used for experiments. Each group of 3–4 mice was used for visualizing and quantifying fluorescent-labeled neurons in three different mouse lines and dendritic spines analysis. Three CRH-IRES-Cre;Ai32 male mice were used for fMOST imaging and neuronal reconstructions. The mice were housed on a 12-h light/dark cycle with food and water provided ad libitum. All animal experiments were performed according to the procedures approved by the Institutional Animal Ethics Committee of the University of Science and Technology of China.

### Histology

All histological procedures have been previously described [26, 53, 54]. Briefly, for whole-brain imaging, mice were anesthetized and perfused with 0.01 M of phosphate-buffered saline (PBS; Sigma-Aldrich Inc., St. Louis, USA), followed by 4% paraformaldehyde (PFA) and 2.5% sucrose in 0.01 M of PBS. The brains were excised and post-fixed in 4% PFA for 24 h. After fixation, each intact brain was rinsed overnight at 4 °C in 0.01 M of PBS and was subsequently dehydrated in a graded ethanol series. Then, the brains were impregnated with glycol methacrylate (GMA, Ted Pella Inc., Redding, CA) and embedded in a vacuum oven.

For immunofluorescence and visualizing fluorescent-labeled neurons in three different mouse lines, the fixed brains were embedded by agarose and consecutive 50- or 100-μm-thick coronal sections were collected using a vibrating microtome (Leica VT1200S, Germany). For immunofluorescence, 50-μm-thick sections were washed three times in PBS (10 min each time) and permeabilized with 0.3% Triton X-100 for 30 min, followed by incubation in 5% normal-donkey-serum blocking solution at room temperature for 1 h. Sections were then incubated with rabbit anti-CRF (1:2000, Bachem, T4037), rabbit anti-MAP 2 (1:200, SYSY, 188002), rabbit anti-Chromogranin B (1:100, Abcam, ab12242), or mouse anti-Kinesin (1:200, Millipore, MAB1614) primary antibody in PBS containing 0.3% Triton X-100 overnight or for 24–36 h at 4 °C. After washing in PBS, sections were incubated with Alexa Fluor 594 or 647-conjugated donkey anti-rabbit or donkey anti-mouse secondary antibody (1:200, Jackson Immuno Research) diluted in 0.1% Triton X-100 in PBS at room temperature for 2 h. After washing in PBS, sections were mounted on slides with antifade mounting medium (Vector Laboratories, Inc., H-1000) and stored at 4 °C. For CRH immunofluorescence, the mice were colchicine pretreated by intracerebroventricular injection of colchicine (0.2 mg/kg) and the samples were collected after 48 h. For visualizing fluorescent-labeled neurons in three different types of mouse lines and dendritic spines, 100-μm-thick sections were washed in PBS and mounted on slides. All images were photographed using an LSM 880 (Zeiss, Germany) or FV3000 (Olympus, Japan) confocal microscope. Abbreviations of brain regions are summarized in Additional file 1, Table S2.

### Whole-brain imaging

Whole-brain imaging was performed by the fMOST system [26]. Briefly, the immersed samples were fixed on the imaging plane, and a WVT system automatically performed the sectioning and imaging to complete the brain-wide data acquisition. We acquired the data sets after sectioning at a 1-μm thickness and imaging at a voxel size of 0.2 × 0.2 × 1 μm or 0.32 × 0.32 × 1 μm. To enhance the in-focus EYFP signal, we added Na_2_CO_3_ into the water bath. Most of the EYFP molecules were preserved in a nonfluorescent state, rather than directly damaged, through chromophore protonation during the resin-embedding procedure. These fluorescent signals were chemically recovered to the fluorescent state using 0.05 M of Na_2_CO_3_ during imaging. For the CRH-IRES-Cre;Ai32 samples, real-time PI staining was performed.

### Image preprocessing

The raw data acquired by the fMOST system required image preprocessing for mosaic stitching and illumination correction. This process has been described previously [26]. Briefly, the mosaics of each coronal section were stitched to obtain an entire section based on accurate spatial orientation and adjacent overlap. Lateral illumination correction was performed section by section. Image preprocessing was implemented in C++ and optimized in parallel using the Intel MPI Library (v.3.2.2.006, Intel). The whole data sets were executed on a computing server (72 cores, 2GHz per core) within 6 h. All full coronal sections were saved at an 8-bit depth in LZW compression TIFF format after image preprocessing.

### Visualization and reconstruction

We visualized data sets using Amira software (v.5.2.2, FEI) and Imaris software (v.9.2.1, bitplane, Switzerland) to generate figures and movies. To process the TB-sized data on a single workstation, we transformed the data format from TIFF to the native LDA type using Amira. The visualization process included extracting the data in the range of interest, sampling or interpolation, reslicing the images, identifying the maximum intensity projection, volume and surface rendering, and generating movies using the main module of Amira. The segmentation editor module of Amira was utilized for the manual outline segmentation of the third ventricle, somata, and varicosities. We applied the filament editor module of Amira to trace the morphologies of EYFP-labeled neurons in 3D via a human-machine interaction. The reconstructed neurons were checked back-to-back by three individuals. The tracing results with original position information were saved in SWC format and the results of the analyses were generated by L-Measure software (v.5.3).

### Statistics

All statistical graphs were generated using GraphPad Prism v.6.01. Two-tailed Student’s *t* tests and one-way ANOVAs, followed by Tukey’s post hoc tests, were also performed using Graphpad Prism v.6.01 and SPSS (IBM SPSS Statistics 23). A *P* < 0.05 was considered to be statistically significant. All results are presented as the mean ± standard error of the mean (SEM).

## Supplementary Information

**Additional file 1:** **Figure S1.** Generation of transgenic mouse lines and morphological features of CRH neurons in the BST and CeA. **Figure S2.** The novel EYFP-labeled CRH neurons identification and high-resolution images showing diverse morphologies of CRH neurons throughout the brains of CRH-IRES-Cre;Ai32 mice. **Figure S3.** Expression specificity and dendritic analysis in the mPFC of CRH-IRES-Cre;Ai32 mice. **Figure S4.** The somatic locations and examples of the reconstructions of somata and dendritic varicosities of reconstructed neurons in the PaAp and Pe and immunofluorescent staining identification of EYFP-labeled CRH neuron and the dendritic varicosities in the CRH-IRES-Cre;Ai32 mice. **Table S1.** Parameters of somatic volume, total dendritic length, and the number of dendritic branches of the reconstructed neurons in several brain regions. **Table S2.** Abbreviation for brain regions.

**Additional file 2: Movie 1.** Movie of serial sections showing the fiber projections from OB.

**Additional file 3: Movie 2.** Movie of serial sections showing the fiber projections from IO.

**Additional file 4: Movie 3.** 3D movie showing the type II and type III connections of CRH neurons in the mPFC.

**Additional file 5.** Excel file for the individual data values used in the Figs. 1 and 3 and the information of all antibodies used.

## Data Availability

All data generated or analyzed during this study are included in this article and its supplementary information files.

## References

[1] Vale W, Spiess J, Rivier C, Rivier J (1981). Characterization of a 41-residue ovine hypothalamic peptide that stimulates secretion of corticotropin and beta-endorphin. Science..

[2] Albeck DS, McKittrick CR, Blanchard DC, Blanchard RJ, Nikulina J, McEwen BS, Sakai RR (1997). Chronic social stress alters levels of corticotropin-releasing factor and arginine vasopressin mRNA in rat brain. J Neurosci.

[3] Mezey E, Kiss JZ, Skirboll LR, Goldstein M, Axelrod J (1984). Increase of corticotropin-releasing factor staining in rat paraventricular nucleus neurones by depletion of hypothalamic adrenaline. Nature..

[4] Miyata M, Okada D, Hashimoto K, Kano M, Ito M (1999). Corticotropin-releasing factor plays a permissive role in cerebellar long-term depression. Neuron..

[5] Puder BA, Papka RE (2001). Distribution and origin of corticotropin-releasing factor-immunoreactive axons in the female rat lumbosacral spinal cord. J Neurosci Res.

[6] Sasaki M, Sato H (2013). Polysynaptic connections between Barrington’s nucleus and sacral preganglionic neurons. Neurosci Res.

[7] Gallopin T, Geoffroy H, Rossier J, Lambolez B (2006). Cortical sources of CRF, NKB, and CCK and their effects on pyramidal cells in the neocortex. Cereb Cortex.

[8] Charlton BG, Ferrier IN, Perry RH (1987). Distribution of corticotropin-releasing factor-like immunoreactivity in human brain. Neuropeptides..

[9] Bassett JL, Foote SL (1992). Distribution of corticotropin-releasing factor-like immunoreactivity in squirrel monkey (Saimiri sciureus) amygdala. J Comp Neurol.

[10] Foote SL, Cha CI (1988). Distribution of corticotropin-releasing-factor-like immunoreactivity in brainstem of two monkey species (Saimiri sciureus and Macaca fascicularis): an immunohistochemical study. J Comp Neurol.

[11] Merchenthaler I (1984). Corticotropin releasing factor (CRF)-like immunoreactivity in the rat central nervous system. Extrahypothalamic distribution. Peptides.

[12] Delville Y, Stires C, Ferris CF (1992). Distribution of corticotropin-releasing hormone immunoreactivity in golden hamster brain. Brain Res Bull.

[13] Cummings SL (1989). Distribution of corticotropin-releasing factor in the cerebellum and precerebellar nuclei of the cat. J Comp Neurol.

[14] Shu YM, Ni RJ, Sun YJ, Fang H, Zhou JN (2015). Distribution of corticotropin-releasing factor in the tree shrew brain. Brain Res.

[15] Wamsteeker Cusulin JI, Fuzesi T, Watts AG, Bains JS (2013). Characterization of corticotropin-releasing hormone neurons in the paraventricular nucleus of the hypothalamus of Crh-IRES-Cre mutant mice. PLoS One.

[16] Itoi K, Talukder AH, Fuse T, Kaneko T, Ozawa R, Sato T, Sugaya T, Uchida K, Yamazaki M, Abe M, Natsume R, Sakimura K (2014). Visualization of corticotropin-releasing factor neurons by fluorescent proteins in the mouse brain and characterization of labeled neurons in the paraventricular nucleus of the hypothalamus. Endocrinology..

[17] Kono J, Konno K, Talukder AH, Fuse T, Abe M, Uchida K, Horio S, Sakimura K, Watanabe M, Itoi K (2017). Distribution of corticotropin-releasing factor neurons in the mouse brain: a study using corticotropin-releasing factor-modified yellow fluorescent protein knock-in mouse. Brain Struct Funct.

[18] Peng J, Long B, Yuan J, Peng X, Ni H, Li X, Gong H, Luo Q, Li A (2017). A quantitative analysis of the distribution of CRH neurons in whole mouse brain. Front Neuroanat.

[19] Chen Y, Molet J, Gunn BG, Ressler K, Baram TZ (2015). Diversity of reporter expression patterns in transgenic mouse lines targeting corticotropin-releasing hormone-expressing neurons. Endocrinology..

[20] De Francesco PN, Valdivia S, Cabral A, Reynaldo M, Raingo J, Sakata I, Osborne-Lawrence S, Zigman JM, Perello M (2015). Neuroanatomical and functional characterization of CRF neurons of the amygdala using a novel transgenic mouse model. Neuroscience..

[21] Huang L, Garcia I, Jen HI, Arenkiel BR (2013). Reciprocal connectivity between mitral cells and external plexiform layer interneurons in the mouse olfactory bulb. Front Neural Circuits.

[22] Garcia I, Bhullar PK, Tepe B, Ortiz-Guzman J, Huang L, Herman AM, Chaboub L, Deneen B, Justice NJ, Arenkiel BR (2016). Local corticotropin releasing hormone (CRH) signals to its receptor CRHR1 during postnatal development of the mouse olfactory bulb. Brain Struct Funct.

[23] Nguyen AQ, Dela Cruz JA, Sun Y, Holmes TC, Xu X (2016). Genetic cell targeting uncovers specific neuronal types and distinct subregions in the bed nucleus of the stria terminalis. J Comp Neurol.

[24] Ragan T, Kadiri LR, Venkataraju KU, Bahlmann K, Sutin J, Taranda J, Arganda-Carreras I, Kim Y, Seung HS, Osten P (2012). Serial two-photon tomography for automated ex vivo mouse brain imaging. Nat Methods.

[25] Gong H, Zeng S, Yan C, Lv X, Yang Z, Xu T, Feng Z, Ding W, Qi X, Li A, Wu J, Luo Q (2013). Continuously tracing brain-wide long-distance axonal projections in mice at a one-micron voxel resolution. Neuroimage..

[26] Gong H, Xu D, Yuan J, Li X, Guo C, Peng J, Li Y, Schwarz LA, Li A, Hu B, Xiong B, Sun Q, Zhang Y, Liu J, Zhong Q, Xu T, Zeng S, Luo Q (2016). High-throughput dual-colour precision imaging for brain-wide connectome with cytoarchitectonic landmarks at the cellular level. Nat Commun.

[27] Zheng T, Yang Z, Li A, Lv X, Zhou Z, Wang X, Qi X, Li S, Luo Q, Gong H, Zeng S (2013). Visualization of brain circuits using two-photon fluorescence micro-optical sectioning tomography. Opt Express.

[28] Alon T, Zhou L, Perez CA, Garfield AS, Friedman JM, Heisler LK (2009). Transgenic mice expressing green fluorescent protein under the control of the corticotropin-releasing hormone promoter. Endocrinology..

[29] Hupalo S, Martin AJ, Green RK, Devilbiss DM, Berridge CW (2019). Prefrontal corticotropin-releasing factor (CRF) neurons act locally to modulate frontostriatal cognition and circuit function. J Neurosci.

[30] Chen P, Lou S, Huang ZH, Wang Z, Shan QH, Wang Y, Yang Y, Li X, Gong H, Jin Y, Zhang Z, Zhou JN (2020). Prefrontal cortex corticotropin-releasing factor neurons control behavioral style selection under challenging situations. Neuron..

[31] Kolber BJ, Boyle MP, Wieczorek L, Kelley CL, Onwuzurike CC, Nettles SA, Vogt SK, Muglia LJ (2010). Transient early-life forebrain corticotropin-releasing hormone elevation causes long-lasting anxiogenic and despair-like changes in mice. J Neurosci.

[32] Lu A, Steiner MA, Whittle N, Vogl AM, Walser SM, Ableitner M, Refojo D, Ekker M, Rubenstein JL, Stalla GK, Singewald N, Holsboer F, Wotjak CT, Wurst W, Deussing JM (2008). Conditional mouse mutants highlight mechanisms of corticotropin-releasing hormone effects on stress-coping behavior. Mol Psychiatry.

[33] Regev L, Tsoory M, Gil S, Chen A (2012). Site-specific genetic manipulation of amygdala corticotropin-releasing factor reveals its imperative role in mediating behavioral response to challenge. Biol Psychiatry.

[34] Stenzel-Poore MP, Heinrichs SC, Rivest S, Koob GF, Vale WW (1994). Overproduction of corticotropin-releasing factor in transgenic mice: a genetic model of anxiogenic behavior. J Neurosci.

[35] Madisen L, Zwingman TA, Sunkin SM, Oh SW, Zariwala HA, Gu H, Ng LL, Palmiter RD, Hawrylycz MJ, Jones AR, Lein ES, Zeng H (2010). A robust and high-throughput Cre reporting and characterization system for the whole mouse brain. Nat Neurosci.

[36] Taniguchi H, He M, Wu P, Kim S, Paik R, Sugino K, Kvitsiani D, Fu Y, Lu J, Lin Y, Miyoshi G, Shima Y, Fishell G, Nelson SB, Huang ZJ (2011). A resource of Cre driver lines for genetic targeting of GABAergic neurons in cerebral cortex. Neuron..

[37] Walker LC, Cornish LC, Lawrence AJ, Campbell EJ (2019). The effect of acute or repeated stress on the corticotropin releasing factor system in the CRH-IRES-Cre mouse: a validation study. Neuropharmacology..

[38] Dedic N, Kuhne C, Jakovcevski M, Hartmann J, Genewsky AJ, Gomes KS, Anderzhanova E, Pohlmann ML, Chang S, Kolarz A, Vogl AM, Dine J, Metzger MW, Schmid B, Almada RC, Ressler KJ, Wotjak CT, Grinevich V, Chen A, Schmidt MV, Wurst W, Refojo D, Deussing JM (2018). Chronic CRH depletion from GABAergic, long-range projection neurons in the extended amygdala reduces dopamine release and increases anxiety. Nat Neurosci.

[39] Park SJH, Pottackal J, Ke JB, Jun NY, Rahmani P, Kim IJ, Singer JH, Demb JB (2018). Convergence and divergence of CRH Amacrine cells in mouse retinal circuitry. J Neurosci.

[40] Swanson LW, Sawchenko PE, Rivier J, Vale WW (1983). Organization of ovine corticotropin-releasing factor immunoreactive cells and fibers in the rat brain: an immunohistochemical study. Neuroendocrinology..

[41] De Souza EB, Insel TR, Perrin MH, Rivier J, Vale WW, Kuhar MJ (1985). Corticotropin-releasing factor receptors are widely distributed within the rat central nervous system: an autoradiographic study. J Neurosci.

[42] Merchenthaler I, Hynes MA, Vigh S, Schally AV, Petrusz P (1984). Corticotropin releasing factor (CRF): origin and course of afferent pathways to the median eminence (ME) of the rat hypothalamus. Neuroendocrinology..

[43] Lewis DA, Foote SL, Cha CI (1989). Corticotropin-releasing factor immunoreactivity in monkey neocortex: an immunohistochemical analysis. J Comp Neurol.

[44] Rho JH, Swanson LW (1989). A morphometric analysis of functionally defined subpopulations of neurons in the paraventricular nucleus of the rat with observations on the effects of colchicine. J Neurosci.

[45] Bittar TP, Nair BB, Kim JS, Chandrasekera D, Sherrington A, Iremonger KJ (2019). Corticosterone mediated functional and structural plasticity in corticotropin-releasing hormone neurons. Neuropharmacology..

[46] Cahusac PM, Castro MG, Robertson L, Lowenstein PR (1998). Electrophysiological evidence against a neurotransmitter role of corticotropin-releasing hormone (CRH) in primary somatosensory cortex. Brain Res.

[47] Yan XX, Baram TZ, Gerth A, Schultz L, Ribak CE (1998). Co-localization of corticotropin-releasing hormone with glutamate decarboxylase and calcium-binding proteins in infant rat neocortical interneurons. Exp Brain Res.

[48] Hupalo S, Bryce CA, Bangasser DA, Berridge CW, Valentino RJ, Floresco SB (2019). Corticotropin-releasing factor (CRF) circuit modulation of cognition and motivation. Neurosci Biobehav Rev.

[49] Hu P, Liu J, Maita I, Kwok C, Gu E, Gergues MM, Kelada F, Phan M, Zhou JN, Swaab DF, Pang Z, Lucassen PJ, Roepke TA, Samuels BA (2020). Chronic stress induces maladaptive behaviors by activating corticotropin-releasing hormone signaling in the mouse oval bed nucleus of the stria terminalis. J Neurosci..

[50] Miklos IH, Kovacs KJ (2002). GABAergic innervation of corticotropin-releasing hormone (CRH)-secreting parvocellular neurons and its plasticity as demonstrated by quantitative immunoelectron microscopy. Neuroscience..

[51] Madisen L, Mao T, Koch H, Zhuo JM, Berenyi A, Fujisawa S, Hsu YW, Garcia AJ, Gu X, Zanella S, Kidney J, Gu H, Mao Y, Hooks BM, Boyden ES, Buzsaki G, Ramirez JM, Jones AR, Svoboda K, Han X, Turner EE, Zeng H (2012). A toolbox of Cre-dependent optogenetic transgenic mice for light-induced activation and silencing. Nat Neurosci.

[52] Tamamaki N, Yanagawa Y, Tomioka R, Miyazaki J, Obata K, Kaneko T (2003). Green fluorescent protein expression and colocalization with calretinin, parvalbumin, and somatostatin in the GAD67-GFP knock-in mouse. J Comp Neurol.

[53] Yang Z, Hu B, Zhang Y, Luo Q, Gong H (2013). Development of a plastic embedding method for large-volume and fluorescent-protein-expressing tissues. PLoS One.

[54] Xiong H, Zhou Z, Zhu M, Lv X, Li A, Li S, Li L, Yang T, Wang S, Yang Z, Xu T, Luo Q, Gong H, Zeng S (2014). Chemical reactivation of quenched fluorescent protein molecules enables resin-embedded fluorescence microimaging. Nat Commun.

