# Supplementary material for: Single-cell morphological characterization of CRH neurons throughout the whole mouse brain
Source: BMC Biol. 2021 Mar 15;19:47. doi: 10.1186/s12915-021-00973-x (PMC7962243; doi:10.1186/s12915-021-00973-x)
Supplement: Supplementary file 1 — Additional file 1: Figure S1. Generation of transgenic mouse lines and morphological features of CRH neurons in the BST and CeA. Figure S2. The novel EYFP-labeled CRH neurons identification and high-resolution images showing diverse morphologies of CRH neurons throughout the brains of CRH-IRES-Cre;Ai32 mice. Figure S3. Expression specificity and dendritic analysis in the mPFC of CRH-IRES-Cre;Ai32 mice. Figure S4. The somatic locations and examples of the reconstructions of somata and dendritic varicosities of reconstructed neurons in the PaAp and Pe and immunofluorescent staining identification of EYFP-labeled CRH neuron and the dendritic varicosities in the CRH-IRES-Cre;Ai32 mice. Table S1. Parameters of somatic volume, total dendritic length, and the number of dendritic branches of the reconstructed neurons in several brain regions. Table S2. Abbreviation for brain regions. [file 12915_2021_973_MOESM1_ESM.docx]

**Supplementary information:**

**Single-cell morphological characterization of CRH neurons throughout the whole mouse brain**

Yu Wang^a,b^, Pu Hu^a,b^, Qinghong Shan^a,b^, Chuan Huang^a,b^, Zhaohuan Huang^a,b^, Peng Chen^a,b^, Anan Li^b,c^, Hui Gong^b,c,^*, and Jiang-Ning Zhou^a,b,^*

* Hui Gong or Jiang-Ning Zhou

**Email:**  huigong@mail.hust.edu.cn or [jnzhou@ustc.edu.cn](mailto:jnzhou@ustc.edu.cn)

**This PDF file includes:**

Figures S1 to S4

Tables S1 to S2

Legends for Movies 1 to 3

**Other supplementary information for this manuscript include the following:**

Additional File 2-4：Movies 1 to 3

**Supplementary information:**


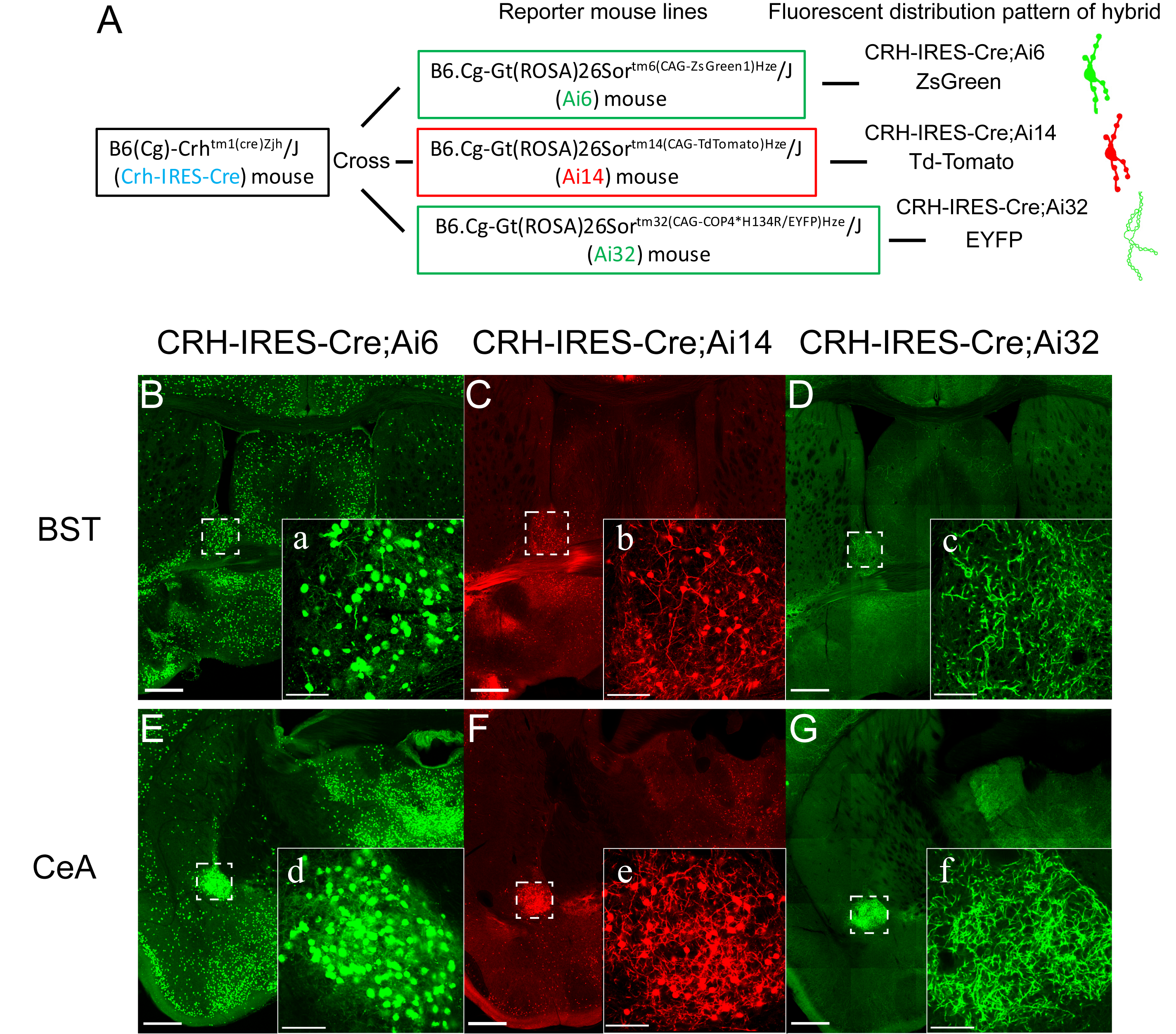


**Fig. S1. Generation of transgenic mouse lines and morphological features of CRH neurons in the BST and CeA.**

(A) Schematic diagram illustrating the generation of three transgenic mouse lines. Breeding CRH-IRES-Cre mice with Ai6, Ai14, and Ai32 mice yielded CRH-IRES-Cre:Ai6, CRH-IRES-Cre:Ai14, and CRH-IRES-Cre:Ai32 mice, in which ZsGreen1, Td-Tomato, and EYFP, respectively, were expressed specifically in cell bodies or fibers of CRH-positive neurons. (B–D) Distributions and morphologies of fluorescent-labeled CRH neurons in the BNST of the three mouse lines. a–c: Magnified images from the dotted boxes in B, C, and D, respectively. (E–G) Distributions and morphologies of fluorescent-labeled CRH neurons in the CeA of the three mouse lines. d–f: Magnified images from the dotted boxes in E, F and G, respectively. Scale bars = 500 μm and 100 μm for the inserts.


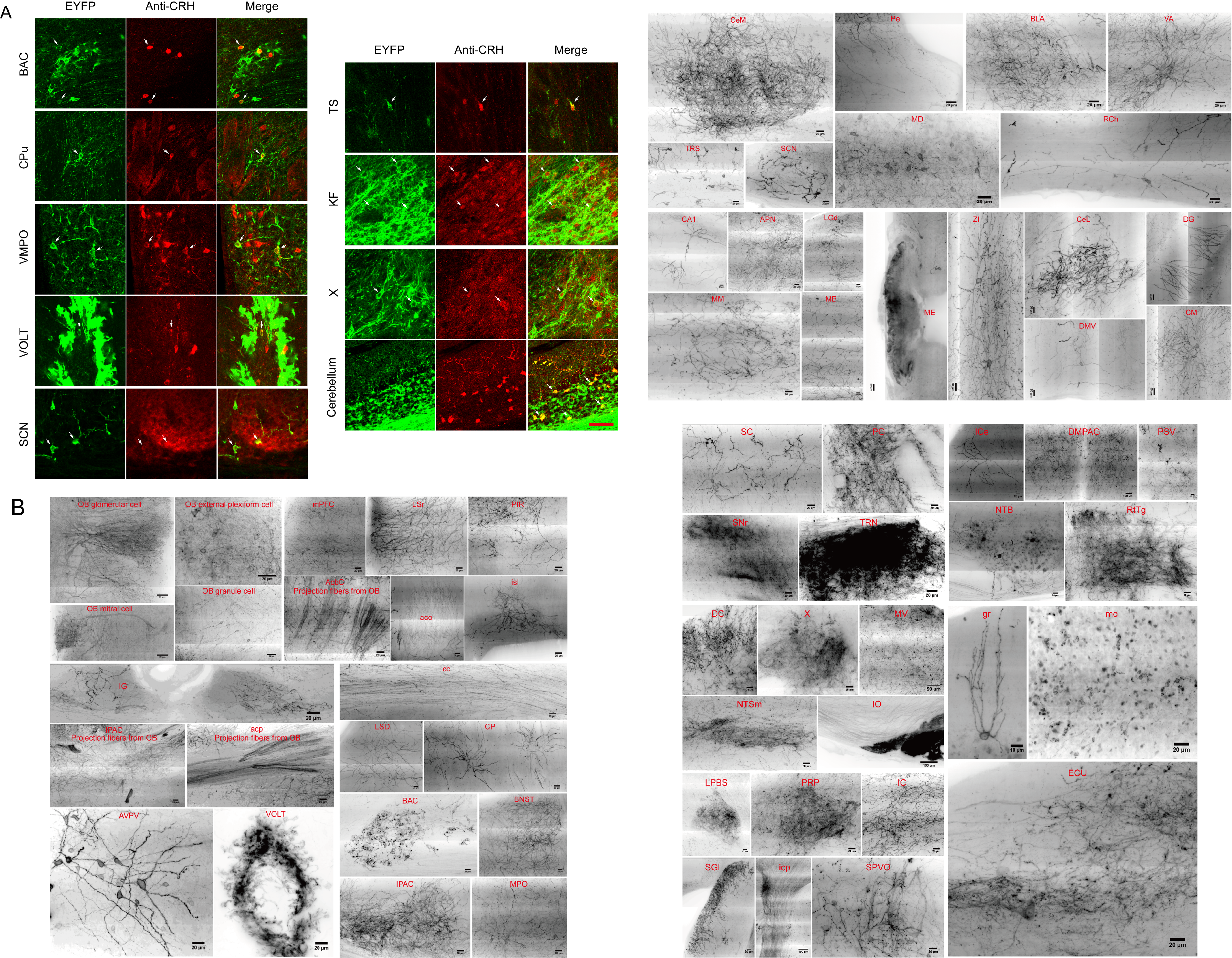


**Fig. S2. The novel EYFP-labeled CRH neurons identification and high-resolution images showing diverse morphologies of CRH neurons throughout the brains of CRH-IRES-Cre;Ai32 mice.**

(A) CRH staining showing the co-labeling of EYFP and CRH immunopositive neurons in several brain regions. The EYFP and CRH immunoreactivity co-labeled cells were indicated by the arrows. Scale bar: 100 μm. (B) High-resolution images showing diverse morphologies of single CRH neurons in different regions.


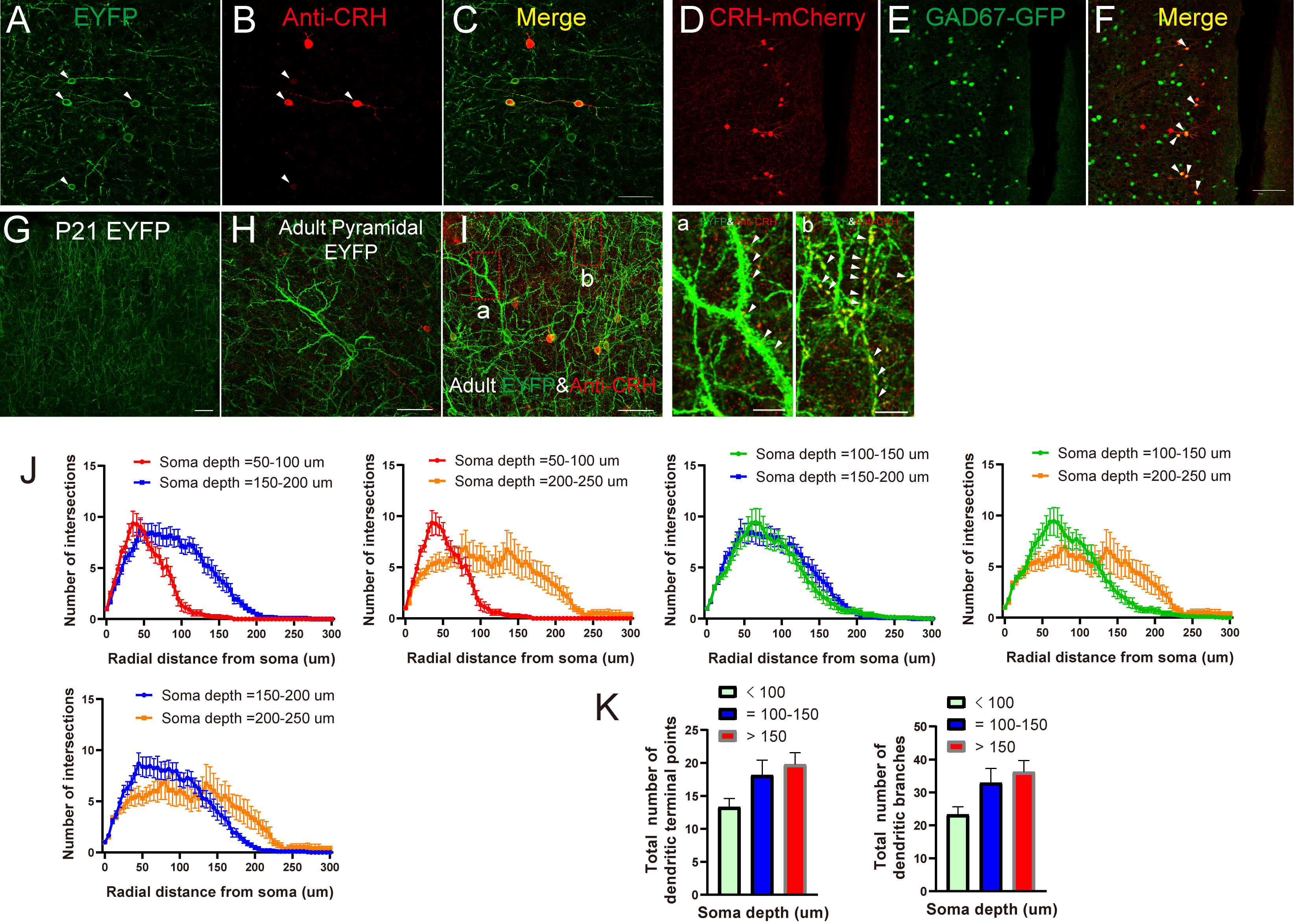


**Fig. S3. Expression specificity and dendritic analysis in the mPFC of CRH-IRES-Cre;Ai32 mice.**

(A–C) Immunofluorescent staining showing that EYFP and CRH immunoreactivity were co-labeled (indicated by the arrowheads) in the mPFC. Scale bar: 50 μm (D–F) CRH-mCherry positive neurons were mainly co-labeled with GAD67-GFP positive cells (indicated by the arrowheads). Scale bar: 100 μm. (G–H) EYFP-labeled pyramidal CRH neurons were visible in adult mice in the mPFC. (I) EYFP-labeled pyramidal neurons included the apical dendrites (red dotted box a) and dendritic spines (indicated by arrowheads in magnified image a) but were not co-labeled with CRH antibodies, while the apical dendrites (red dotted box b) and dendritic swellings (indicated by arrowheads in magnified image b) were CRH immunopositive. Scale bars: G–I: 50 μm, a–b: 10 μm. (J) Sholl analysis of dendrites of neurons with different somatic depths illustrating changes in the mean number of intersections with increasing radial distance from the soma, n = 15 cells for somatic depths = 50–100 μm, 21 cells for somatic depths = 150–200 μm and 10 cells for somatic depths = 200–250 μm. (K) The total number of dendritic terminal points and total number of dendritic branches were not significantly different among neurons with somatic depths < 100 μm, soma depths = 100–150 μm, and somatic depths > 150 μm, n = 13 cells for somatic depths < 100 μm, 21 cells for 100–150 μm and 31 cells for >150 μm.


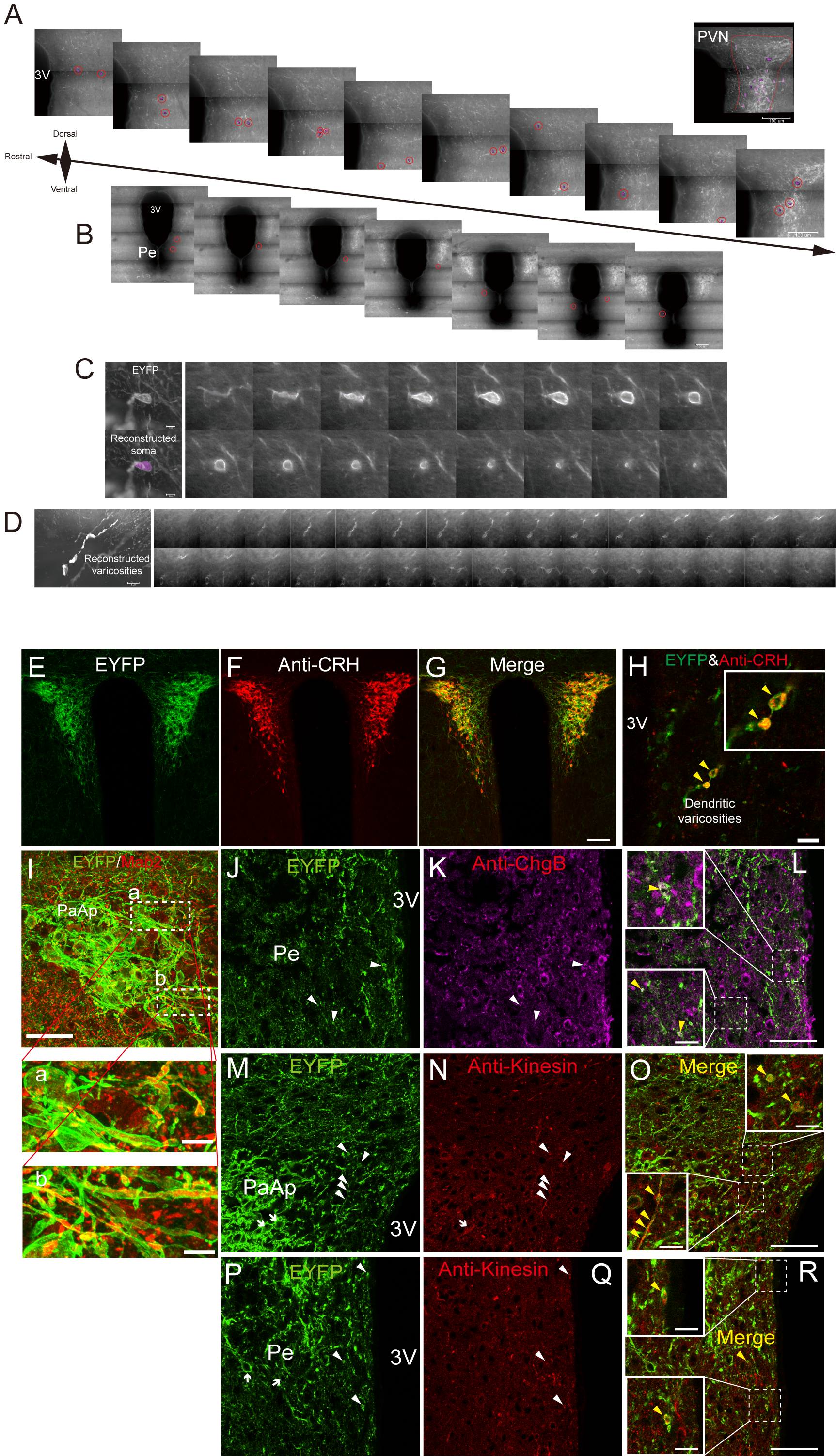


**Fig. S4. The somatic locations and examples of the reconstructions of somata and dendritic varicosities of reconstructed neurons in the PaAp and Pe and immunofluorescent staining identification of EYFP-labeled CRH neuron and the dendritic varicosities in the CRH-IRES-Cre;Ai32 mice.**

(A) The somatic locations (indicated by red circles) of each reconstructed neuron in the PaAp. (B) The somatic locations (indicated by red circles) of each reconstructed neuron in the Pe. (C–D) 3D images and consecutive 1-μm sections showing the reconstruction process of somata (C) and dendritic varicosities (D). (E–G) Immunofluorescent staining showing that EYFP-labeled cells and varicosities co-labeled with CRH immunoreactivity in the PaAp. Scale bar: 100 μm. (I, a and b) EYFP-labeled structures contained MAP2 immunopositive signals in PaAp. Scale bars: 50 μm for I and 10 μm for a and b. (J–L) EYFP-labeled varicosities contained ChgB immunopositive signals (indicated by the yellow arrowheads) in Pe. (M–R) EYFP-labeled varicosities contained Kinesin immunopositive signals (indicated by the yellow arrowheads) in PaAp (M–O) and Pe (P–R). Scale bars: 50 μm and 10 μm for inserts.


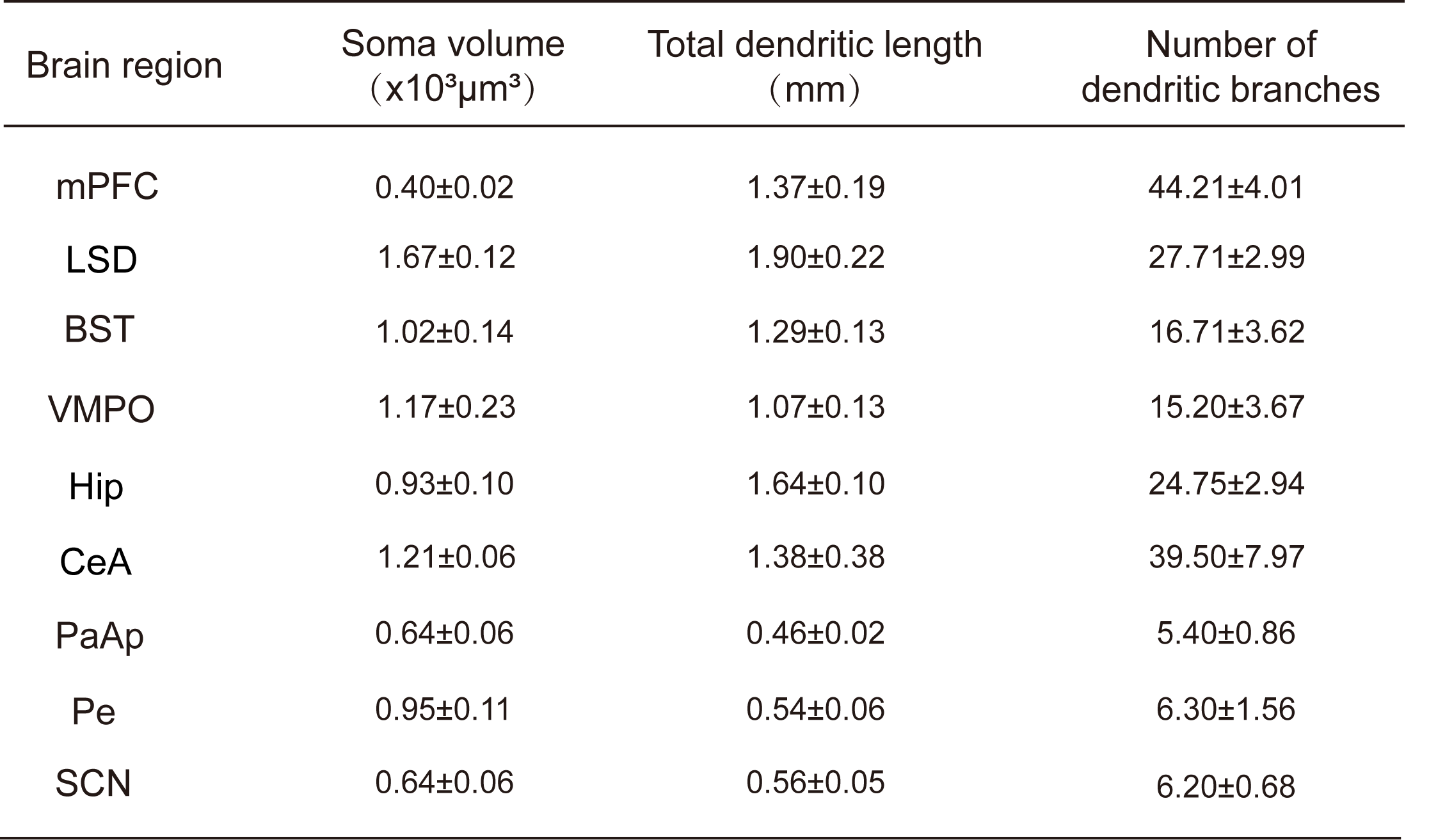


**Table S1. Parameters of somatic volume, total dendritic length, and the number of dendritic branches of the reconstructed neurons in several brain regions.**

**Table S2.** **Abbreviation for brain regions.**

| 3V | third ventricle | icp | inferior cerebellar peduncle | PR | prerubral field |
| --- | --- | --- | --- | --- | --- |
| AcbSh | accumbens nucleus,shell | IG | indusium griseum | PSV | principal sensory nucleus of trigeminal |
| acp | anterior commissure, posterior | IL | infralimbic cortex | Pr5 | principal sensory trigeminal nucleus |
| aco | anterior commissure, olfactory limb | IO | inferior olive | PRP | nucleus prepositus |
| AD | anterodorsal thalamic nucleus | IPAC | interstitial nucleus of the posterior limb of the anterior commissure | PrL | prelimbic cortex |
| AM | anteromedial thalamic nucleus | isl | islands of calleja | PVN | paraventricular hypothalamic nucleus |
| APN | Anterior pretectal nucleus | KF | Kolliker-Fuse nucleus | RCh | retrochiasmatic area |
| APTD | anterior pretectal nucleus, dorsal part | LD | laterodorsal thalamic nucleus | RSG | retrosplenial granular cortex |
| Au1 | primary auditory cortex | LH | lateral hypothalamic area | RtTg: | reticulotegmental nucleus of the pons |
| AuD | secondary auditory cortex, dorsal area | LGd | dorsal part of the lateral geniculate complex | S1 | primary somatosensory cortex |
| AVPV | anteroventral periventricular nucleus | LPBs | lateral parabrachial nucleus, superior part | S2 | secondary somatosensory cortex |
| BAC | bed nucleus of the anterior commissure | LS | lateral septal nucleus | SC | superior colliculus |
| BLA | basolateral amygdaloid nucleus, anterior part | LSD | lateral septal nucleus, dorsal part | SCN | suprachiasmatic nucleus |
| BNST | bed nucleus of the stria terminalis | LSr | lateral septal nucleus, rostral part | SGl | superficial glial zone of the cochlear nuclei |
| CA1 | field CA1 of hippocampus | M1 | primary motor cortex | SPVO | spinal nucleus of the trigeminal, oral part |
| cc | corpus callosum | M2 | secondary motor cortex | SNC | substantia nigra, compact part |
| CeL | central amygdaloid nucleus, lateral division | MB | midbrain | SNr | substantia nigra, reticular part |
| CeM | central amygdaloid nucleus, medial division | MD | mediodorsal thalamic nucleus | SuG | superficial gray layer of the superior colliculus |
| Cg1 | cingulate cortex, area 1 | ME | median eminence | TRN | tegmental reticular nucleus |
| CM | central medial thalamic nucleus | Mi | mitral cell layer | TRS | triangular septal nucleus |
| CP | caudate putamen | MGV | medial geniculate nucleus, ventral part | V1 | primary visual cortex |
| DC | dorsal cochlear nucleus | MM | medial mammillary nucleus, medial part | V2 | secondary visual cortex |
| DG | dentate gyrus | MnR | median raphe nucleus | VA | ventral anterior thalamic nucleus |
| DLG | dorsal lateral geniculate nucleus | MV | medial vestibular nucleus | VCA | ventral cochlear nucleus, anterior part |
| DM | dorsomedial hypothalamic nucleus | mo | molecular layer of the cerebellar cortex | VCP | ventral cochlear nucleus, posterior part |
| DMPAG | dorsomedial periaqueductal gray | MPO | media preoptic area | VLG | ventral lateral geniculate nucleus |
| DMV | dorsomedial hypothalamic nucleus, ventral part | NTB | nucleus of the trapezoid body | VLTg | ventrolateral tegmental area |
| DTT | dorsal tenia tecta | NTSm | nucleus of the solitary tract, medial part | VMPO | ventromedial preoptic nucleus |
| ECU | external cuneate nucleus | OB | olfactory bulb | VOLT | vascular organ of the lamina terminalis |
| EPl | external plexiform layer | PaAp | paraventricular hypothalamic nucleus, anterior parvicellular part | X | nucleus X |
| Gl | glomerular layer | Pe | periventricular hypothalamic nucleus | ZI | zona incerta |
| gr | granular layer of the cerebellar cortex | PF | parafascicular thalamic nucleus |  |  |
| GrO | granule cell layer | PG | pontine gray |  |  |
| Hip | hippocampus | PIR | piriform cortex |  |  |
| IC | inferior colliculus | Pn | pontine nuclei |  |  |
| ICe | inferior colliculus, external nucleus | Po | posterior thalamic nuclear group |  |  |

**Movie 1. Movie of serial sections showing the fiber projections from OB.**

**Movie 2. Movie of serial sections showing the fiber projections from IO.**

**Movie 3. 3 D movie showing the type II and type III connections of CRH neurons in the mPFC.**
